# Hydro‐Functional Traits and Their Dissimilarity to the Neighbourhood Buffer Tree Growth Against the 2018–2020 Central European Drought

**DOI:** 10.1111/gcb.70588

**Published:** 2025-11-13

**Authors:** Lena Sachsenmaier, Florian Schnabel, Fon R. Tezeh, Pablo Castro Sánchez‐Bermejo, Nico Eisenhauer, Olga Ferlian, Sylvia Haider, Ronny Richter, Sharath S. Paligi, Bernhard Schuldt, Christian Wirth

**Affiliations:** ^1^ German Centre for Integrative Biodiversity Research (iDiv) Halle‐Jena‐Leipzig Leipzig Germany; ^2^ Systematic Botany and Functional Biodiversity Leipzig University Leipzig Germany; ^3^ Chair of Silviculture, Faculty of Environment and Natural Resources University of Freiburg Freiburg Germany; ^4^ Chair of Forest Botany Technical University of Dresden Dresden Germany; ^5^ Geobotany and Botanical Garden Martin Luther University Halle‐Wittenberg Halle (Saale) Germany; ^6^ Institute of Ecology Leuphana University of Lüneburg Lüneburg Germany; ^7^ Institute of Biology Leipzig University Leipzig Germany; ^8^ Plant Ecology and Ecosystems Research University of Göttingen Göttingen Germany; ^9^ Max‐Planck Institute for Biogeochemistry Jena Germany

**Keywords:** biodiversity‐ecosystem functioning, climate change, forest, hydraulic safety, neighbourhood, stomatal control, trait dissimilarity, tree diversity, TreeDivNet

## Abstract

Climate change is predicted to increase the frequency and duration of drought events, threatening the functioning of Central European forests. While diverse forests often promote long‐term growth stability, their performance during extreme drought events remains debated. Understanding the effects of forest diversity on tree growth during drought requires not only a consideration of tree interactions with direct neighbouring trees but also of the species' morphological and physiological characteristics, i.e., a trait‐based approach. Contrasting species‐specific drought responses might be driven by hydro‐functional traits, which shape a tree's hydraulic safety and stomatal control strategy. We investigated individual tree growth before, during, and after the unprecedented 2018–2020 Central European drought, from a hydro‐functional perspective. We analysed annual growth data from 2611 trees in a temperate tree diversity experiment (MyDiv experiment, Germany), measured 14 hydro‐functional traits, and modelled individual tree growth across the years 2016–2021, considering the interaction of climatic conditions with hydro‐functional trait syndromes. Our results reveal that traits related to hydraulic safety and stomatal control potential were key drivers of tree growth across drought and non‐drought years. During the severe multi‐year drought, the growth of focal trees increased with either higher hydraulic safety or tighter stomatal control potential. Trait syndromes that were less advantageous under normal conditions provided beneficial effects under drought stress, reflecting a trade‐off in performance across conditions. Additionally, we found that hydro‐functional dissimilarity between a tree and its surrounding neighbors provided benefits for tree growth during drought. Therefore, our study suggests that planting tree mixtures with distinct hydro‐functional strategies can enhance resistance to future droughts.

## Graphical abstract

Climate change is putting forests at risk. Our research shows that a tree's water‐use strategy largely determines its growth across years: whereas one strategy performs better in normal years, the other is advantageous during drought. Trees grow best during drought when surrounded by neighbors whose water‐use strategies differ from their own, suggesting that planting a mix of species with different water‐use strategies could help forests better withstand future droughts.

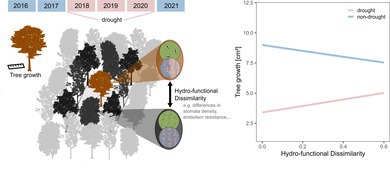

## ZUSAMMENFASSUNG

Der Klimawandel wird Prognosen zufolge die Häufigkeit und Dauer von Dürreereignissen erhöhen und damit die Funktionsfähigkeit mitteleuropäischer Wälder gefährden. Während artenreiche Wälder oft die langfristige Stabilität des Wachstums fördern, bleibt ihre Leistungsfähigkeit bei extremen Dürreereignissen umstritten. Um die Auswirkungen der Walddiversität auf das Wachstum der Bäume bei Trockenheit zu verstehen, müssen nicht nur die Interaktionen der Bäume mit ihren direkten Nachbarn berücksichtigt werden, sondern auch die morphologischen und physiologischen Merkmale der Arten, d.h. ein merkmalsbasierter Ansatz ist daher entscheidend. Unterschiedliche artspezifische Reaktionen auf Trockenheit könnten auf hydrofunktionale Merkmale zurückzuführen sein, die die Strategien der hydraulischen Sicherheit und der stomatären Kontrolle eines Baumes bestimmen. Wir untersuchten das Wachstum einzelner Bäume vor, während und nach der außergewöhnlichen Dürre von 2018–2020 in Mitteleuropa aus einer hydrofunktionalen Perspektive. Wir analysierten jährliche Wachstumsdaten von 2.611 Bäumen in einem Diversitätsexperiment mit temperaten Baumarten (MyDiv‐Experiment, Deutschland), erfassten 14 hydrofunktionale Merkmale und modellierten das individuelle Baumwachstum über die Jahre 2016–2021, wobei wir die Interaktion von klimatischen Bedingungen mit hydrofunktionalen Merkmalssyndromen berücksichtigten. Unsere Ergebnisse zeigen, dass Merkmale, die mit der hydraulischen Sicherheit und dem stomatären Kontrollpotenzial zusammenhängen, wichtige Triebkräfte für das Wachstum der Bäume sowohl in Dürrejahren als auch in Jahren ohne Dürre waren. Während der mehrjährigen extremen Dürre nahm das Wachstum der Bäume mit höherer hydraulischer Sicherheit oder stärkerem stomatären Kontrollpotenzial zu. Merkmalssyndrome, die unter normalen Bedingungen weniger vorteilhaft waren, wirkten sich unter Trockenheitsstress positiv aus, was einen Trade‐off der Leistungsfähigkeit bei unterschiedlichen Bedingungen widerspiegelt. Darüber hinaus haben wir festgestellt, dass die hydrofunktionelle Dissimilarität zwischen einem Baum und seinen Nachbarn das Wachstum der Bäume bei Trockenheit begünstigt. Unsere Studie legt nahe, dass die Pflanzung von Baumarten mit unterschiedlichen hydrofunktionalen Strategien die Widerstandsfähigkeit von Wäldern gegenüber zukünftigen Dürren erhöhen kann.

## 1 Introduction

Climate change leads to more frequent and longer‐lasting drought events, such as the unprecedented 2018–2020 drought in Central Europe, which resulted from consecutive years of low precipitation and unusually high temperatures under persistent anticyclonic circulation (Buras et al. 2020; Hari et al. 2020; Markonis et al. 2021; Rakovec et al. 2022; Zscheischler et al. 2020; Zscheischler and Fischer 2020). This extreme drought led to severe consequences across vast forest areas, such as elevated tree mortality, deterioration of forest health, and strong growth reductions (Bastos et al. 2020, 2021; Haberstroh et al. 2022; Obladen et al. 2021; Pohl et al. 2023; Schuldt et al. 2020; Senf and Seidl 2021). However, the severity of these impacts varied depending on tree species and forest composition (Kretz et al. 2025; Obladen et al. 2021; Schnabel et al. 2022; Thom et al. 2023). Therefore, it is crucial to understand which characteristics enable tree species to withstand extreme drought, such as the 2018–2020 Central European drought, and whether this resistance depends on the functional composition of neighbouring trees within a forest stand.

Mixed forests are often recommended for their potential to enhance long‐term productivity and stability (Fichtner et al. 2018; Messier et al. 2022; Schnabel et al. 2019). However, the role of tree diversity in reducing vulnerability to drought remains debated and appears to be context‐dependent (Grossiord 2020; Serrano‐León et al. 2025). Recent findings suggest that while diverse forests can help to buffer against mild drought (Schnabel et al. 2021, 2024), their effectiveness may diminish during extreme droughts (Haberstroh and Werner 2022). This potential shift from positive to neutral or even negative diversity effects under extreme conditions highlights the need to better understand how tree diversity influences drought responses. Studies in experimental tree plantations investigating the 2018–2020 drought found that tree diversity alone may not be sufficient to protect forest stands from the negative effects of severe drought on tree growth (Sachsenmaier et al. 2024; Shovon et al. 2024). Instead, species identity and species composition of forest stands were found to play critical roles in shaping productivity during drought (Decarsin et al. 2024; Hajek et al. 2022; Sachsenmaier et al. 2024).

Such species‐specific responses to drought can be attributed to trait‐based mechanisms, particularly those regulating water use (Anderegg et al. 2016; Blackman et al. 2019; Choat et al. 2018). Given the complexity of water‐regulation strategies, a whole‐tree perspective integrating all organs (Hartmann et al. 2021; Martínez‐Vilalta et al. 2023) and a broad set of functional traits (*sensu* Violle et al. 2007), is essential for elucidating the drivers of species‐specific drought responses (Waite et al. 2024). These traits are typically categorised into those related to the water‐transport system (hydraulic traits) and those involved in the regulation of transpiration (stomatal traits), henceforth collectively referred to as ‘hydro‐functional traits’.

During drought, some species keep their stomata open and continue transpiring, i.e., they rely on continued water uptake despite a higher risk of xylem embolism (‘drought‐tolerating’), while others reduce their stomatal conductance to minimise transpiration losses and prevent xylem embolism, though at the cost of reduced photosynthetic yield (‘drought‐avoiding’) (Choat et al. 2012). Rather than discrete categories, these strategies form a continuum from loose to tight water potential control, reflecting anisohydric to isohydric behaviour: some species allow water potentials to decline with soil drying, maintaining gas exchange longer, while others maintain stable water potentials through early stomatal closure. (Klein 2014; Martínez‐Vilalta and Garcia‐Forner 2017; McDowell et al. 2008).

The stringency of stomatal control can be approximated by stomatal conductance during drought periods. Morphological traits like stomatal density and size, often inversely related (de Boer et al. 2016; Liu et al. 2023), can serve as useful proxies: species with higher stomatal density and smaller guard cells tend to regulate transpiration more rapidly and precisely, offering an advantage in retaining water under drought conditions, whereas species with fewer, larger stomata may lose water more quickly under high evaporative demand (Drake et al. 2013; Kröber and Bruelheide 2014; Wang et al. 2015). Water potential dynamics further reflect these strategies: a limited decline in midday water potential despite decreasing predawn values suggests tighter stomatal regulation, while a simultaneous drop in both midday and predawn water potentials indicates looser control (Fu and Meinzer 2019; Meinzer et al. 2016; Waite et al. 2024). Next to stomatal control, another key dimension of drought resistance is the resistance of the xylem against embolism formation, often quantified by the water potential at which 50% of xylem conductivity is lost (P_50_) (Brodribb 2009; Choat et al. 2012, 2018). The minimum water potential a species experiences complements this and can indicate how close a species typically operates to its hydraulic limits under drought (Delzon and Cochard 2014; Meinzer et al. 2009). Traits of the leaf economics spectrum (LES), which reflect conservative versus acquisitive resource‐use strategies (Reich 2014; Wright et al. 2004), were found to correlate with the gradient of xylem embolism resistance (Kröber et al. 2014; Schnabel et al. 2021). Considering LES traits, such as specific leaf area (SLA) and leaf dry matter content (LDMC), may thus help to link drought tolerance to broader ecological strategies.

Since a precise understanding of how these hydro‐functional traits and trait syndromes (i.e., coordinated sets of traits that reflect a common strategy) are linked to each other remains incomplete, a comprehensive set of hydro‐functional traits should be considered for a solid characterization of tree species strategies during drought (see Table S1). Recent studies in subtropical systems identified two orthogonal axes of trait variation, mainly representing traits related to stomatal control and embolism resistance, respectively, as fundamental to a tree's drought response strategy (Kröber et al. 2014; Schnabel et al. 2021, 2024). Whether these patterns hold across different contexts and how they relate to tree performance during severe drought events remain open questions.

Further, a tree's growth may be determined not just by its own functional trait identity, but also by the functional composition of its neighbourhood, defined by the traits of its surrounding trees. Whether a tree can benefit or not from its neighbours during drought was found to be highly species‐specific. Trees that were more sensitive to drought tended to enhance the growth or survival of less affected neighbouring trees (Fichtner et al. 2020; Hajek et al. 2022; Lübbe et al. 2017; Paligi et al. 2024; Schnabel et al. 2024). Besides, potential resource partitioning or facilitation between dissimilar tree neighbours could explain such beneficial effects (Grossiord 2020). Resource partitioning occurs when species use different water or nutrient sources in space or time, thereby reducing competition, whereas facilitation involves positive interactions in which one tree improves the conditions for another, for instance, through hydraulic redistribution (i.e., passive water transfer from deep to shallow soil layers) (Neumann and Cardon 2012; Prieto et al. 2012; Zou et al. 2005), ameliorating microclimatic conditions via shading, or by limiting herbivore damage (Jactel and Brockerhoff 2007). Given that a focal trees' hydro‐functional traits may affect growth differently during drought versus non‐drought years, a tree neighbourhood may benefit more from maintaining high diversity in these traits and thus different response strategies to variable climatic conditions, rather than from a uniform trait expression in one single direction.

Recent studies have demonstrated that the diversity in drought‐tolerance traits enhances the stability of forest community growth under interannual climatic variability (Gazol and Camarero 2016; Schnabel et al. 2021). Focusing on the neighbourhood scale, where tree‐tree interactions occur (Trogisch et al. 2021), is particularly informative, as it allows the examination of diversity effects in conjunction with other factors crucial to focal tree growth, such as tree size and the competition with or mortality of direct neighbour trees (Fichtner et al. 2020; Forrester and Pretzsch 2015; Schnabel et al. 2024). While the overall trait diversity in a neighbourhood captures a broad spectrum of functional strategies, it does not directly reflect how a focal tree interacts with its neighbours based on how its traits compare to those of a specific neighbour (Fortunel et al. 2016). During drought, such functional dissimilarity may shape whether interactions are competitive or complementary: trees with similar neighbours may face stronger competition, while dissimilarity can reduce it (Nemetschek et al. 2025).

**FIGURE 1:**
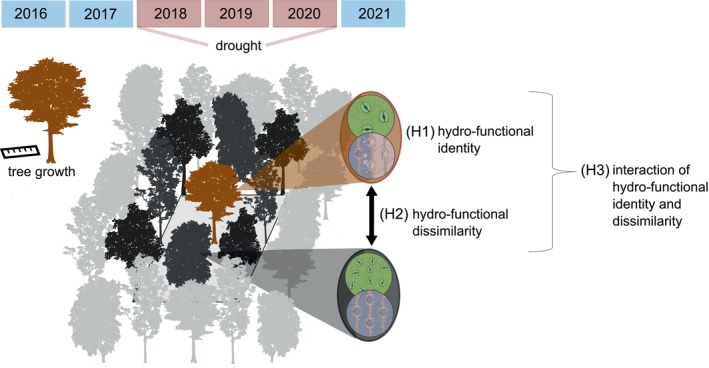
Conceptual framework of the study, linking tree growth during drought to hydro‐functional traits and their dissimilarity to neighbouring trees. Focal tree growth between 2016 and 2021 was analysed in relation to (H1) the focal tree's hydro‐functional identity, (H2) its hydro‐functional dissimilarity to neighbouring trees and (H3) the interaction of both effects.

In light of this, understanding how functional dissimilarity to a tree neighbourhood influences growth during severe drought can provide key insights into how forests maintain their functionality in the face of climate change. Here, we focus on how individual tree growth is shaped by the interactive effects of drought, the tree's hydro‐functional identity, and its hydro‐functional dissimilarity to the neighbourhood (see Figure 1). Specifically, we test (H1) whether the hydro‐functional trait identity of trees explains growth differently in drought years versus non‐drought years, (H2) whether greater hydro‐functional dissimilarity of the focal tree to its neighbourhood increases its growth during drought years compared to growing in more hydro‐functionally similar neighbourhoods, and (H3) whether a tree's hydro‐functional identity determines the relationship between its hydro‐functional dissimilarity to the neighbourhood and its growth.

## 2 Materials and Methods

### 2.1 Study Site and Experimental Design

This study was conducted at the MyDiv tree diversity experiment in central Germany, at the Bad Lauchstädt Experimental Research Station of the Helmholtz Centre for Environmental Research–UFZ (51°23′ N, 11°53′ E; 118 m a.s.l.). The site is characterised by a continental climate (484 mm annual precipitation, 8.8°C mean annual air temperature, 1896–2003; see Figure S1), with Haplic Chernozem soil developed from loess (Altermann et al. 2005). Established in 2015 on former agricultural land, the experiment includes 80 plots (11 × 11 m), each planted with 140 tree saplings in a 1 m grid (Ferlian et al. 2018). Ten common temperate deciduous tree species were planted in 20 monocultures (two replicates per species), 30 two‐species mixtures, and 30 four‐species mixtures ( *Acer pseudoplatanus* L., *Aesculus hippocastanum* L., *Betula pendula* Roth, *Carpinus betulus* L., *Fagus sylvatica* L., *Fraxinus excelsior* L., *Prunus avium* (L.) L., *Quercus petraea* (Matt.) Liebl., *Sorbus aucuparia* L., *Tilia platyphyllos* Scop.) (for details see Tables S2 and S3). Within each plot, we designated the 36 innermost trees in a 6 × 6 m area as *focal trees*, for a total of up to 2880 individual trees. Each focal tree is typically surrounded by eight neighbouring trees, with four at a 1 m distance and four at a 1.41 m distance, which define the *focal tree's neighbourhood* (see Figure 1). Given the small variation in spatial distances, which is unlikely to result in meaningful differences in competitive influence among the neighbours, we considered all eight neighbouring trees equally in subsequent analyses.

### 2.2 Identification of Drought

We defined drought years as years with increased water deficits compared to long‐term averages of meteorological parameters (Schwarz et al. 2020). To determine drought years at our site, we employed the Standardized Precipitation Evapotranspiration Index (SPEI; Vicente‐Serrano et al. (2010)), based on data derived from the closest weather station to the site (DWD Climate Data Center, [CDC], Station Leipzig/Halle, ID 2932; Figures S2 and S3). SPEI series were computed for a 40‐year reference period (1982–2022) with the SPEI package (Beguería et al. 2014) in R (R Core Team 2023) using monthly precipitation and potential evapotranspiration data (via the Penman‐Monteith equation (Allen et al. 1998)). We classified years as normal, particularly dry (SPEI ≤ −1), or particularly wet (SPEI ≥ +1). The years 2018, 2019 and 2020 were consistently classified as three consecutive drought years, regardless of the accumulation period considered (SPEI‐3, SPEI‐6, SPEI‐12). Based on the 6‐month SPEI (SPEI‐6) during the vegetation period (April–September), 2018 and 2019 fell into the category of severe drought (values of −1.9 and −1.6, respectively), while 2020 was a moderate drought year (−1.3). On‐site soil moisture data collected since 2017 confirmed the pronounced drought conditions during 2018–2020 (Figure S4; see Sachsenmaier et al. (2024) for details).

### 2.3 Tree Growth Responses

From 2015 to 2021, stem diameters at 5 cm above the ground (basal diameter, *d*0; cm) were recorded for all individual trees across the 80 plots using a diameter tape, with measurements taken annually in winter (December–January). To correct for measurement errors (5.2% of all values) caused by inconsistent positions or tree structure, we estimated *d*0 using individual‐based allometric models that predicted *d*0 from diameter at breast height (DBH) and tree height, improving accuracy where direct measurements were unreliable. Trees with incomplete data series and from a damaged monoculture plot were excluded (see Data S1 for details on this data cleaning procedure).

To determine annual growth, we calculated the tree basal area increment (BAI) for each tree and year based on the basal stem diameter (*d*0):

(1) $${\mathrm{BAI}}_{\text{year}}=\pi \;{\left(\frac{d0_{\text{year}}}{2}\right)}^{2}-\pi \;{\left(\frac{d0_{\text{year}-1}}{2}\right)}^{2}$$

where *d*0_year_ represents the tree's diameter in the current year and *d*0_year‐1_ its diameter from the previous year. The final dataset consists of the growth of 2611 tree individuals over 6 years, corresponding to 15,666 data points (for details on trees per species, see Table S2).

### 2.4 Functional‐Trait Measurements

The measurement of the 14 different functional traits is described briefly below, with full details provided in the Data S2.

#### 2.4.1 Embolism Resistance

Measurements were conducted on sun‐exposed canopy branches sampled from monoculture plots. We quantified species‐specific embolism resistance (P_50_) using Cavitron‐based vulnerability curves for diffuse‐porous species (Cochard et al. 2005) and the optical method at the leaf level using cavicams for ring‐porous species (Brodribb et al. 2016; Gauthey et al. 2020). Due to methodological artefacts, P_50_ values for *F. excelsior* were taken from a parallel study in a nearby forest. To avoid artefacts associated with maximum vessel length (Martin‐StPaul et al. 2014; Torres‐Ruiz et al. 2017), different techniques and organs were used to construct vulnerability curves (branches using centrifuge for diffuse‐porous species vs. leaves using the optical method for ring‐porous species). Previous comparisons have shown good agreement between these approaches (Brodribb et al. 2017); however, absolute values should be interpreted with some caution, as organ‐ and method‐specific differences may exist (Venturas et al. 2019).

#### 2.4.2 Xylem Anatomy and Wood Density

We measured vessel density (VD) and hydraulically weighted vessel diameter (Dh) from stained branch cross‐sections using light microscopy and image analysis. Wood density (WD) was calculated as dry mass divided by fresh volume using the water displacement method.

#### 2.4.3 Stomatal Density and Stomatal Size

Stomatal density (stomata mm^−2^) and guard cell length (μm) were measured on nail polish imprints taken from the abaxial surface of sun‐exposed canopy leaves in monocultures, using light microscopy at 400× and 1000× magnifications, respectively.

#### 2.4.4 Leaf Water Potentials and Hydroscape Area

We measured predawn and midday leaf water potentials (Ψ_PD_ and Ψ_MD_) during a summer drought period in 2022 (Figure S5) to assess species‐specific water potential dynamics. Measurements were taken on healthy, sun‐exposed canopy leaves using a pressure chamber Scholander et al. (1965). Ψ_MD_ was used as a proxy for minimum leaf water potential (Ψ_min_), and the differences between midday and predawn water potentials (ΔΨ) were calculated to assess water loss throughout the day. To assess a species' degree of isohydry, we estimated hydroscape areas (HSA) as the area between the Ψ_PD_–Ψ_MD_ regression and the 1:1 line (Meinzer et al. 2016), with larger values indicating greater flexibility in stomatal regulation across changing water availability.

#### 2.4.5 Stomatal Conductance

We measured stomatal conductance (*g*_s_) on sun‐exposed upper canopy leaves of the same tree individuals used for water potential measurements, using a hand‐held porometer (LI‐600, LI‐COR, Lincoln, Nebraska USA) under clear‐sky conditions. Repeated measurements were taken across the day (6:30 AM–3:00 PM) to capture diurnal variation during drought (Figure S5). The mean *g*_s_ across all measurement points was used as *g*_s (drought)_, reflecting each species' ability to maintain transpiration under drought stress. We additionally estimated residual conductance (*g*_res_) as the mean *g*_s_ at vapor pressure deficit (VPD) > 3 kPa, which reflects extreme dry conditions at which we assume stomata to be closed.

#### 2.4.6 Leaf Carbon Isotopes

At the end of the 2022 drought year's growing season, we sampled leaves from the upper canopy of three randomly selected trees per monoculture plot. Leaf carbon isotope discrimination (δ^13^C, ‰) was determined using an elemental analyser connected to an isotope ratio mass spectrometer (IRMS) at the BGC‐IsoLab (MPI for Biogeochemistry, Jena, Germany). δ^13^C was calculated relative to the international VPDB standard, serving as an indicator of intrinsic water‐use efficiency (Farquhar et al. 1989).

#### 2.4.7 Specific Leaf Area and Leaf Dry Matter Content

In August 2021, we collected fully developed, healthy leaves from 16 trees per species in monoculture plots (five leaves per tree at different canopy heights). Leaf spectra were measured with visible–near infrared reflectance spectrometry (Vis‐NIRS). Specific leaf area (SLA; leaf area divided by leaf dry mass) and leaf dry matter content (LDMC; leaf dry mass divided by leaf fresh mass) were predicted using a convolutional neural network approach (Vasseur et al. 2022), trained on a calibration set of leaves measured with standard laboratory protocols. Model validation yielded *R* ^2^ values of 0.88 for SLA and 0.86 for LDMC. We used this predictive approach instead of direct measurements to ensure methodological consistency with a previous study at the site. For further details on sampling, analytical procedures and predictive ability of the method, see Castro Sánchez‐Bermejo et al. (2024).

All traits were measured on trees grown in monoculture to characterise species‐specific functional strategies in the absence of confounding effects of interspecific interactions. While trait expressions may vary in response to intra‐ and interspecific interactions in mixed stands (Castro Sánchez‐Bermejo et al. 2024), species‐level trait values provide a robust approximation of intrinsic functional strategies. Importantly, even when some traits were measured under drought conditions, all species were assessed under the same environmental context, so the relative differences among species remain meaningful and reflect inherent functional strategies.

### 2.5 Axes of Trait Variation

For all 14 measured traits, we calculated the species mean values (Table S4), standardised them (z‐transformation), and used them to perform a Principal Components Analysis (PCA). The PCA was performed with the *prcomp()* function in R. With this, we reduced the dimensionality of the trait dataset by identifying orthogonal axes (principal components, PCs) that explain the largest variance in the data, with each axis representing a combination of trait loadings. These axes were interpreted and named based on their associated trait loadings. We used Horn's parallel analysis, as implemented in the *paran* package (Dinno 2024), to determine the number of components to retain from the PCA. The analysis, based on the eigencomposition of the correlation matrix (420 random iterations), identified two components with adjusted eigenvalues greater than 1 (2.43 for PC1 and 1.31 for PC2), which were retained for further analysis. We used the scores of the first two principal components (PC1 and PC2) for each species, as continuous variables to represent their hydro‐functional strategies in the subsequent analyses.

### 2.6 Quantification of Hydro‐Functional Dissimilarity

To assess the functional dissimilarity of individual focal trees to their neighbourhood, we used a modified version of the standard functional dispersion metric (FDis), provided by the *FD* package (Laliberté et al. 2023). First, we calculated pairwise functional distances between species using the *gowdis()* function of the FD package (Laliberté et al. 2023), which implements Gower's (1971) dissimilarity. For continuous traits, *gowdis()* internally scales each trait by its observed range when computing pairwise differences, such that the trait‐level distance between species *j* and *k* for trait *i* is:

(2) $$d_{\mathrm{ijk}}=\frac{|\;x_{\mathrm{ij}}-x_{\mathrm{ik}}\;|}{\;x_{i_{\max}}-x_{i_{\min}}}$$

These trait‐level distances are then combined across all 14 traits to produce a single species‐level functional distance matrix ranging from 0 (identical species) to 1 (maximally dissimilar species; Table S5).

For each focal tree, we calculated its functional dissimilarity to the surrounding neighbourhood (FDissim) as the weighted mean of these functional distances to neighbouring species, using their basal area as weights (reflecting the assumption that larger neighbours have a stronger influence):

(3) $$\text{FDissim}=\frac{\sum_{i=1}^{n}\left(\mathrm{fun}\_{\text{distance}}_{i}\times {\text{size}}_{i}\right)}{\sum_{i=1}^{n}\left(\;{\text{size}}_{i}\right)}$$

where fun_distance_*i*_ represents the functional distance between focal tree species and the neighbouring species_*i*_ (see distance matrix Table S5), size_*i*_ is the basal area of the neighbouring tree, and *n* is the number of neighbouring trees (up to eight per focal tree). The calculations were performed iteratively for each focal tree per year using custom functions in R. The neighbouring trees were not weighted according to their spatial distance to the focal tree, because the distance variation (1.0 m vs. 1.41 m) is much less likely to substantially influence competitive interactions compared to the highly biologically relevant size differences between neighbours (up to ~300 cm^2^); adding a second weighting factor for spatial distance would not have been computationally feasible within the framework of our iterative calculations.

We applied the same method to calculate dissimilarity along PC1 and PC2 axes by using species' PC scores instead of the 14 trait means (see Table S6). Note that we excluded monocultures from all FDissim analyses, as the neighbourhood dissimilarity would always be zero by design.

### 2.7 Statistical Analysis

We used linear mixed‐effects models (LMMs) to examine the effects of hydro‐functional strategies and functional dissimilarity to the neighbouring trees on individual tree growth (as basal area increment) during the years 2016–2021. In all models, we accounted for the size of the focal tree (its basal area) and its competition with neighbours, calculated as the sum of the basal area of all surrounding trees larger than the focal tree (both variables log‐transformed and scaled). We accounted for our experimental design and repeated measurements by a nested random‐effects structure of the identity of the focal tree nested within the plot. We used a logarithmic transformation for basal area increment, adding a constant (+37) to all values beforehand to account for negative values. For an overview of all models performed, please see Table S7.

First, we examined how the hydro‐functional traits of the focal trees affected individual tree growth responses during the years 2016–2021. We built two models, using either PC1 or PC2 scores (in interaction with year) as the main predictors. Second, we examined how a focal tree's dissimilarity to its neighbourhood in terms of hydro‐functional traits affected individual tree growth during the years 2016–2021. We built three models, each with one of the following main predictors: dissimilarity in all hydro‐functional traits, dissimilarity on the PC1 axis, and dissimilarity on the PC2 axis (all in interaction with year). Year‐specific effects were evaluated using the *emtrends()* function from the *emmeans* package (Lenth 2023), producing standardized effect sizes (SES) to quantify the strength of these relationships across years. Third, we tested whether the focal tree's hydro‐functional identity modulates the effect of its dissimilarity from the neighbourhood on its growth during the years 2016–2021. We built two separate models, both with the three‐way interaction between dissimilarity, year and hydro‐functional strategy (PC1 or PC2). Even though the PC scores were modelled as continuous variables, for clarity and ease of interpretation in the results, we predicted effects for the values of 1 and −1 as representative of high and low extremes of each strategy. To interpret the interaction, we estimated simple slopes of FDissim at high and low values of PC1 and PC2 (defined as ±1 standard deviation from the mean) separately for each year using the *emtrends()* function from the *emmeans* package (Lenth 2023). Pairwise comparisons of these slopes were performed using the *contrast()* function to determine whether the effect of FDissim differed significantly between high and low hydraulic safety or high and low stomatal control conditions, respectively.

Model assumptions such as normality, homoscedasticity and linearity of residuals were checked visually using diagnostic plots of the *performance package* (Lüdecke et al. 2021). All analyses were performed in R version 4.4.1 (R Core Team 2023) using the *lme4* package (Bates et al. 2015) and the *lmerTest* package (Kuznetsova et al. 2017) for linear mixed‐effects models, the *emmeans* package (Lenth 2023) to extract model results and conduct post hoc tests and the *tidyverse* environment for data processing and graphical plotting (Wickham et al. 2019).

## 3 Results

### 3.1 Hydro‐Functional Trait Coordination

**FIGURE 2:**
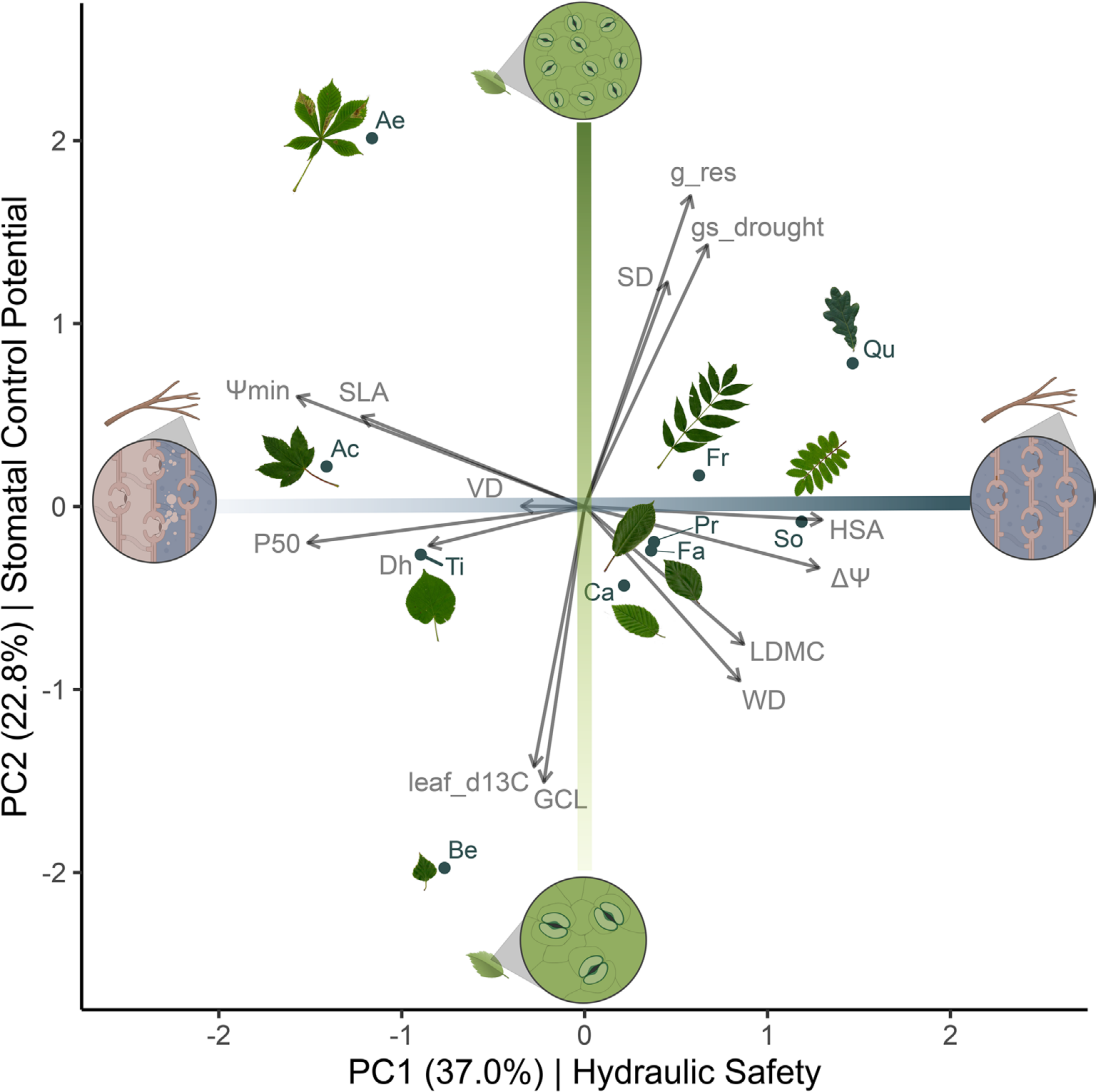
Main axes of hydro‐functional trait variation. Biplot of the first two axes of a Principal Component Analysis (PCA) based on 14 hydro‐functional traits in 10 temperate tree species. The analysis uses species mean trait values obtained from trees growing in monoculture plots within a temperate tree diversity experiment (MyDiv; Ferlian et al. (2018)). Leaf silhouettes represent the tree species, with genus abbreviations indicated (Ac = *Acer pseudoplatanus* , Ae = *Aesculus hippocastanum* , Be = *Betula pendula* , Ca = *Carpinus betulus* , Fa = *Fagus sylvatica* , Fr = *Fraxinus excelsior* , Pr = *Prunus avium* , Qu = *Quercus petraea* , So = *Sorbus aucuparia* , Ti = *Tilia platyphyllos* ). PC1 reflects a hydraulic safety gradient running from hydraulic vulnerable water transport systems (low embolism resistance (less negative P_50_), less negative minimum leaf water potential (Ψmin), high specific leaf area (SLA)) to hydraulic safer systems (high hydroscape area (HSA), large leaf water potential difference (ΔΨ), high leaf dry matter content (LDMC), high wood density (WD)). PC2 reflects a stomatal control potential gradient running from species with low control potential (large guard cell length (GCL), high carbon isotope discrimination in a drought year) to species with high control potential (high stomatal density (SD), high stomatal conductance during drought and residual conductance (gs_drought, g_res)). The illustrations visualise the trait gradients. For details on PCA please see Table S8.

Using 14 hydro‐functional traits of 10 tree species, we found that the first two axes of a principal component analysis (PCA) together explained 59.8% of the total variation in our dataset (Figure 2).

The first principal component axis (PC1) was associated with hydroscape area (HSA), water potential differences between midday and predawn (ΔΨ), minimum water potential (Ψ_min_), embolism resistance (P_50_), leaf dry matter content (LDMC), branch wood density (WD) and specific leaf area (SLA), in descending order of contribution (see Figure S6a, and Table S8 for loadings). This axis thus reflected a gradient from low to high hydraulic resistance, aligned with the transition from an acquisitive resource strategy (high SLA) to a conservative resource strategy (high LDMC, high wood density). It is hereafter called the ‘Hydraulic Safety’ axis, explaining 37% of the total variance in trait space. The second principal component axis (PC2) was primarily associated with the residual leaf conductance during drought (*g*_res_), guard cell length (GCL), leaf carbon isotope discrimination (δ^13^C), stomatal conductance during drought (*g*_s (drought)_), and stomatal density (SD), in descending order of contribution (see Figure S6b, and Table S8 for loadings). This axis represents a gradient from large stomata, low stomatal density, and high carbon isotope discrimination to small stomata, high stomatal density, and increased stomatal and residual conductance during drought. It explained 22.8% of the total variance and is hereafter referred to as the ‘Stomatal Control Potential’ axis. The third PC axis captured a trade‐off between vessel density and size, explaining 15.8% of the variance (Figure S7), but was excluded based on Horn's parallel test, which identified only the first two axes as meaningful (see Methods).

### 3.2 The Effect of Hydro‐Functional Traits on Tree Growth

**FIGURE 3:**
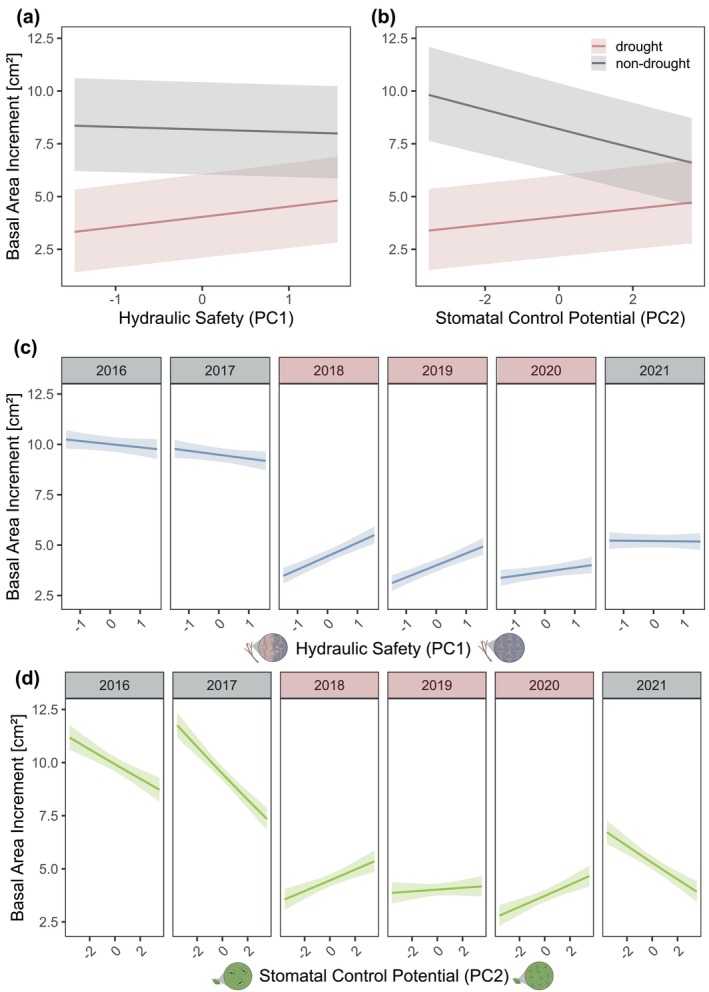
The effect of Hydro‐functional Identity (hydraulic safety, stomatal control potential) on tree growth across years. The focal tree's basal area increment (BAI, log‐transformed) was predicted by its functional traits related to (a, c) hydraulic safety and (b, d) stomatal control potential across years, using linear mixed‐effects models. Tree size and competition were included as additional fixed effects; random effects account for tree ID nested in plot. Regression lines and 95% confidence intervals reflect model fits. The Hydraulic Safety model (c) explained 55% (marginal *R* ^2^) and 59% (conditional *R* ^2^) of the variation in BAI; the Stomatal Control Potential model (d) explained 56% and 59%, respectively. See Tables S9 and S10 for full model results.

Overall, we found a clear decline in growth rates during the three drought years 2018–2020 (Figure 3). We found the axes of hydraulic safety and stomatal control potential to significantly predict changes in annual growth. Notably, the direction of the relationship shifted depending on the environmental conditions, i.e., between drought and non‐drought years (Figure 3a,b). The interaction between hydraulic safety and year significantly influenced tree growth (*F*_5,12,869_ = 18.7; *p* < 0.001; Table S9). Under normal conditions in the years 2016, 2017, and 2021, having a hydraulically safe or non‐safe system appeared to be less important for growth. However, during the drought years 2018–2020, this trait spectrum became important, with species possessing safer hydraulic systems showing higher growth compared to others (Figure 3c). A similar pattern was observed for stomatal control potential, which significantly predicted tree growth in interaction with year (*F*_5,12,841_ = 45.2; *p* < 0.001; Table S10). In non‐drought years, species with a lower stomatal control potential displayed substantially higher growth. In contrast, during drought, having a higher stomatal control potential enhanced growth significantly (Figure 3d).

### 3.3 The Effect of Hydro‐Functional Dissimilarity on Tree Growth

**FIGURE 4:**
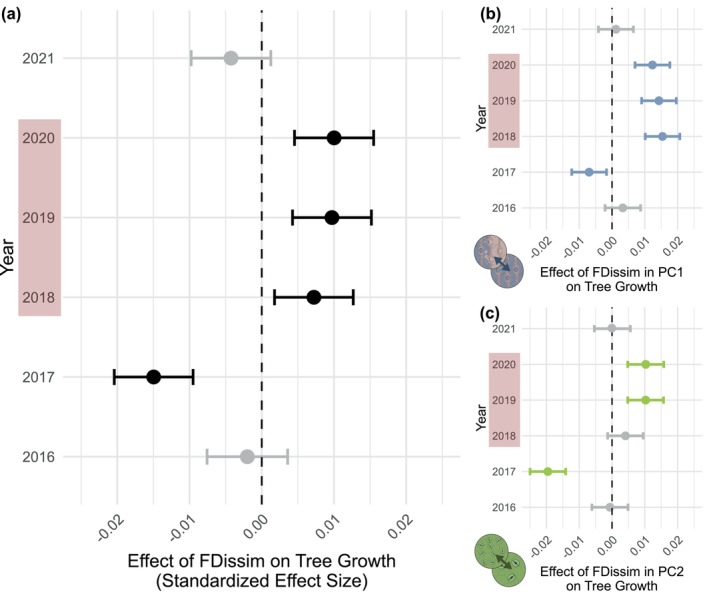
Standardized effect sizes (SES) of Hydro‐functional Dissimilarity (FDissim) on tree growth across years. (a) Overall, FDissim across all hydro‐functional traits, (b), FDissim in hydraulic safety (PC1) and (c) FDissim in stomatal control potential (PC2) based on linear mixed‐effects models. Tree size and competition were included as additional fixed effect; random effects account for tree ID nested in plot. Error bars represent 95% confidence intervals. Vertical dashed lines indicate zero effects. Significant effects (CI excluding zero) are displayed in black or colour, non‐significant effects are shown in grey. Negative SES indicates a negative relationship between FDissim and tree growth; positive SES indicates a positive one. Drought years (2018–2020) are shaded red. The interaction between FDissim and year was significant (*p* < 0.05) in all models, with explained variance (conditional/marginal *R* ^2^) of (a) 61.2%/57.5%, (b) 61.4%/57.5%, and (c) 61.2%/57.5%.

Functional dissimilarity in hydro‐functional traits was a significant predictor of tree growth across the study years (*F*_5,9760_ = 17.59; *p* < 0.001; Table S11). During the 2018–2020 drought, trees with greater dissimilarity to their neighbouring trees showed significantly higher growth rates, with the strength of this effect slightly increasing over the three years (Figure 4a). In contrast, no significant effects were found in non‐drought years 2016 and 2021, and a negative effect occurred in 2017, reflecting overall contrasting patterns between drought and non‐drought conditions (Figure S8).

When separating dissimilarity into hydraulic safety (PC1) and stomatal control potential (PC2), both remained significant predictors of growth (PC1: *F*_5,9763_ = 14.3; *p* < 0.001; PC2: *F*_5,9759_ = 22.3; *p* < 0.001). For hydraulic safety, greater dissimilarity consistently increased growth during drought, though the effect slightly declined with increasing drought severity (Figure 4b). For stomatal control potential, positive effects appeared in 2019 and 2020 but not yet in 2018 (Figure 4c). Comparing the effect sizes, dissimilarity regarding hydraulic safety more strongly promoted growth during drought than dissimilarity in stomatal control potential (Tables S12 and S13).

**FIGURE 5:**
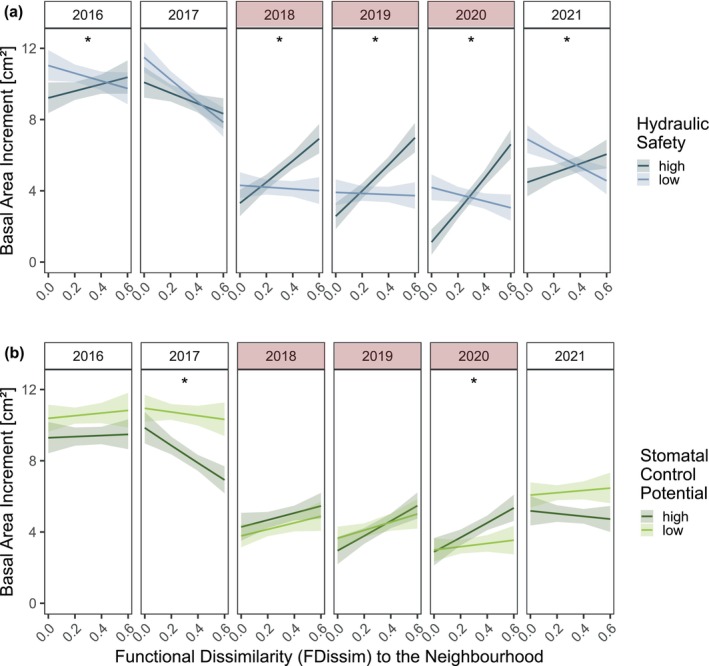
Interactive effects of Functional Dissimilarity to the Neighbourhood (FDissim) and Functional Identity (hydraulic safety/stomatal control potential) on tree growth across years. Regression lines and confidence intervals (95% CIs) show linear mixed‐effects model fits of basal area increment (log‐transformed), based on the interactive effects of FDissim, year and hydraulic safety (a) or stomatal control potential (b). Tree size and competition were included as additional fixed effects; random effects account for tree ID nested in plot. Both models explained 58% and 61% of growth variation through their fixed (marginal *R* ^2^) and fixed and random effects (conditional *R* ^2^). Asterisks indicate years where slopes differ significantly between high and low trait groups. See Tables S14 and S15 for model results and Tables S16–S18 for pairwise slope comparisons.

By considering the interactive effects of functional identity and functional dissimilarity, we could identify which functional strategy expressions benefited from high functional dissimilarity to their neighbourhood (Figure 5, Tables S14 and S15). Along the hydraulic safety axis, species with high hydraulic safety displayed increased growth when surrounded by functionally dissimilar neighbours in almost all years, but in particular during the drought years (Figure 5a, Figure S9a). In contrast, species with low hydraulic safety experienced reduced growth in the presence of highly dissimilar neighbours under non‐drought conditions. However, during drought, this negative effect of functional dissimilarity was no longer apparent (Table S16). Except in 2017, the two groups consistently showed significantly different slopes (Table S18), implying distinct responses to functional dissimilarity in their neighbourhood.

Examining the stomatal control potential axis, we found that during drought functional dissimilarity was generally beneficial for both strategic expressions, regardless of whether high or low stomatal control strategy (Figure 5b, Figure S9b). In the drought year 2019, this positive effect of dissimilarity was strongest and significant for both stomatal control expressions. In the drought year 2020, the high stomatal control potential group still benefited from functional dissimilarity, while the low stomatal control potential group did not anymore (Table S17). In non‐drought years, we did not find any significant effect of functional dissimilarity on tree growth, except in 2017, when dissimilarity had a significant negative effect on species with high stomatal control potential, with no effect for the low stomatal control potential group. Only in the years 2017 and 2020, did the two stomatal control potential groups display significantly different slopes (Table S18).

## 4 Discussion

The strong growth reductions we observed during the 2018–2020 drought align with previous findings from the same trees at the community level (Sachsenmaier et al. 2024) and in Central Europe in general (Schnabel et al. 2022; Schuldt et al. 2020; Thom et al. 2023). During the severe, multi‐year drought, the growth of the focal trees increased with either higher hydraulic safety or tighter stomatal control potential. However, these two hydro‐functional strategies, which appear to be beneficial under drought, had either opposite or non‐significant effects on tree growth in non‐drought years. In addition, only under drought conditions, hydro‐functional dissimilarity to the trees in the immediate neighbourhood creates a growth advantage that disappeared in non‐drought years. These results highlight the context‐dependency of hydro‐functional trait effects on tree growth and can guide management decisions for future, drought‐adapted forests.

### 4.1 Hydraulic Safety and Stomatal Control Potential Form Distinct Trait Syndromes

Considering the species mean trait values of 14 hydro‐functional traits, we identified two orthogonal axes that represent gradients of hydraulic safety and stomatal control potential (Figure 2). The clear orthogonality suggests that there is no close coupling between the two trait syndromes of hydraulic safety and stomatal control potential. These may operate on different temporal dimensions: stomatal regulation may be most critical from the onset of drought until stomatal closure, whereas hydraulic safety becomes the dominant factor afterwards (Blackman et al. 2016; Martin‐StPaul et al. 2017; Waite et al. 2024). We expected, in particular, HSA to be relevant to both PCA axes or, as in other studies, to be more closely associated with stomatal stringency (Waite et al. 2024). However, the pattern we found may be explained by the exceptional drought conditions during the measurements, suggesting that when stomatal regulation reaches its limits, the prevention of large fluctuations in water potential (high ΔΨ and HSA) depends more on xylem traits. Beyond xylem hydraulics, additional features such as rooting depth or water storage capacity are also important for the regulation of plant water potential and can be broadly viewed as drought avoidance mechanisms (Liang and Ye 2024). Indeed, our results from PC1 indicate a negative correlation between P50 and both ΔΨ and HSA, suggesting a trade‐off between drought avoidance (maintaining higher water potential through tight regulation, or access to deep soil water or internal water storage) and tolerance strategies (tolerating lower water potentials through high xylem resistance). Importantly, the exclusion of ΔΨ and HSA did not change the orthogonality of hydraulic safety and stomatal control potential in our study (see the PCA without isohydry traits in Figure S10).

On the hydraulic safety gradient, species with a high ΔΨ and HSA were associated with rather conservative strategies, possibly delaying stomatal closure and relying on either high xylem resistance (Brodribb et al. 2003; Klein 2014) or on belowground systems like a more extended root system or higher fine root acclimation potential (Jaeger et al. 2024; Kahmen et al. 2022). Notably, we found that the midday leaf water potential values in 2022 were quite close to the respective P_50_ for some species (e.g., *B. pendula* with a branch P_50_: −2.19 MPa and a leaf Ψmin: −1.85 MPa, see Figures S11 and S12 for more details), indicating that the occurrence of substantial embolism during the severe drought we studied is quite likely for these species. We thus assume that embolism occurred in 2018, 2019 and 2020. We found SLA, LDMC, and wood density to align with the hydraulic safety axis (Figure 2). This means that high structural investments not only contribute to longer‐lasting tissues and more efficient resource use but also enhance resistance to embolism and thus improve longevity in stress situations such as drought.

In our study, the stomatal control potential axis reflects a gradient from large stomata and high carbon isotope discrimination to high stomatal density, and increased stomatal and residual conductance during drought (Figure 2). As shown in previous studies, species with smaller but denser stomata (de Boer et al. 2016; Liu et al. 2023) exhibited faster and more precise transpiration control (Drake et al. 2013; Durand et al. 2019; Kröber and Bruelheide 2014; Lawson and Vialet‐Chabrand 2019; Wang et al. 2015). However, a faster stomatal response not only means quicker closure under intensifying atmospheric dryness but also enables tighter control for water loss during drought, such as selectively keeping stomata open in favorable leaf areas, like near large veins (Lawson and Blatt 2014; Mott and Buckley 1998). This might explain why, in our study, which took place during particularly dry conditions, only species with high stomatal density also displayed considerable stomatal conductance during drought (Henry et al. 2019). For an in‐depth discussion of the trait coordination results, see Data S3. Despite incorporating multiple traits related to stomata, it is important to note that the stomatal control potential gradient in our PCA was largely driven by two species with notably differing values: *A. hippocastanum* at the upper end and *B. pendula* at the lower end (Figure 2). Further, a broad generalization about stomatal control strategies remains challenging, as studies often rely on different traits to describe this gradient. The existence of universal trait syndromes that define forest responses to drought remains a debated topic (Leuschner et al. 2019; Martínez‐Vilalta and Garcia‐Forner 2017; Oliveira et al. 2021; Waite et al. 2024) and requires further research.

### 4.2 Hydro‐Functional Traits Drive Opposing Growth Patterns in Drought and Non‐Drought Years

We found that both the hydraulic safety strategy and the stomatal control strategy strongly explained tree growth (Figure 3). During drought, species with safer hydraulic systems and tighter stomatal control potential were associated with increased growth, whereas in non‐drought years, these strategies were linked to lower growth. This finding confirms our hypothesis (H1), which posits that hydro‐functional trait identity influences growth differently under drought and non‐drought conditions. In the absence of drought, a species' hydraulic safety strategy had mostly a negative impact on growth, likely due to the safety–efficiency trade‐off, where embolism‐resistant xylem tends to have lower water transport efficiency and thus limits growth under favorable conditions (Fan et al. 2011; Hajek et al. 2014; Hoeber et al. 2014). Conversely, when water availability is limited under drought conditions and the risk of xylem embolism increases, a safe hydraulic system becomes advantageous, as reflected in its positive relationship with tree growth during drought years. The hydraulic safety–efficiency trade‐off, typically observed across species (Torres‐Ruiz et al. 2024) helps to explain why species with highly embolism‐resistant xylem can sustain lower water potentials and maintain stomatal conductance and photosynthesis despite prolonged drought, ultimately leading to greater cumulative growth over the growing season. The strongest effect of hydraulic safety on growth occurred in 2018, the most severe drought year, followed by 2019 and 2020, suggesting its importance increases with drought severity. The severe drought may have caused embolisms in species with fragile xylem, and the resulting xylem dysfunction or costly repair investment (Klein et al. 2018; Meinzer and McCulloh 2013; Ogasa et al. 2013) could explain their reduced growth.

Previous studies found that acquisitive species often suffer the greatest growth reductions during drought (Fichtner et al. 2020; Schnabel et al. 2024), as observed in genera such as *Betula*, *Salix* and *Populus* during the 2018–2020 drought (Schuldt et al. 2020). Given that in our study, as well as in former studies (e.g., Schnabel et al. (2024)), the acquisitive‐conservative trade‐off is inherently linked to the hydraulic safety trade‐off examined, it is conceivable that the strong growth reductions in acquisitive species are driven by their low embolism resistance. Conservative species, in contrast, may benefit from shaded positions in early successional stages, reducing evaporative demand and buffering them against drought stress (Bennett et al. 2015; McGregor et al. 2021). Although taller trees face greater canopy water stress due to a higher hydrostatic gradient and the longer travel path (Fernández‐de‐Uña et al. 2023), we found the hydraulic safety gradient to not closely match current tree size. For example, *A. hippocastanum* falls on the acquisitive end of the gradient, yet remains one of the smallest species (Dietrich et al. 2022; Sachsenmaier et al. 2024), aligning with emerging evidence that acquisitive tree species do not necessarily achieve fast growth (Augusto et al. 2025).

Species with larger stomata showed lower stomatal conductance during drought and higher leaf δ^13^C after a drought year (Figure 3b), suggesting prolonged stomatal closure and limited photosynthesis. This may have led to carbon reserve depletion and reduced growth (Stefaniak et al. 2024). In contrast, species with smaller and denser stomata maintained stomatal opening and transpiration, sustaining photosynthesis and likely supporting higher growth during drought. Continued transpiration can also cool leaves, preventing overheating, protecting photosynthetic function and avoiding cell membrane damage (Berry and Bjorkman 1980; Blasini et al. 2022). Notably, Marchin et al. (2022) found that during a heatwave, plants increased stomatal conductance even past turgor loss points when growing in dry soils. Since the 2018–2020 drought was accompanied by heatwaves (Bastos et al. 2021; Knutzen et al. 2025), similar processes may have operated at our site, potentially explaining the positive link between stomatal control potential and growth—through sustained transpiration, despite or even because of high temperatures. Alternatively, increased residual conductance after heat exposure could reflect thermal damage to leaf cuticles (Fernandes et al. 2025). In non‐drought years, we found species with larger stomata showing greater growth (Figure 3b), likely due to higher conductance and larger volumes of water for carbon assimilation, assuming sufficient xylem efficiency to support this uptake.

Our study design assigned fixed hydraulic safety and stomatal control potential strategies to each species across years, without accounting for potential shifts in water‐use strategies (Özçelik and Poyatos 2025), phenotypic plasticity, or acclimatisation to drought (Awad et al. 2010; Corcuera et al. 2011; Niemczyk et al. 2024; Petrik et al. 2022). Even though some drought‐related trait adjustments have been reported in general (Rowland et al. 2023), their magnitude and timing are likely insufficient to explain the consistent patterns already observed in the first drought year, 2018.

### 4.3 Hydro‐Functional Dissimilarity to the Tree Neighbourhood Improves Tree Growth During Drought

We found that hydro‐functional dissimilarity to the tree neighbourhood had a positive effect on tree growth, but only during drought (Figure 4). This finding supports our hypothesis (H2) and aligns with the idea that positive species interactions become more critical under challenging climatic conditions (Bertness and Callaway 1994; Hisano et al. 2018), without being able to disentangle whether these effects arise from complementarity (niche differences reducing competition), facilitation (active positive interactions), or both. The consistently significant positive impact of hydro‐functional dissimilarity across all three drought years is particularly noteworthy.

Even though previous studies have already demonstrated that diversity in water‐related traits within a neighbourhood can mitigate drought effects (Anderegg et al. 2018; Fichtner et al. 2020; Hajek et al. 2024; Schnabel et al. 2021, 2024), it remained unclear whether these responses would simply become more pronounced or whether they would fundamentally change under severe drought events. Several studies suggest that tree diversity per se may not buffer communities against the impacts of severe drought (Blondeel et al. 2024; Decarsin et al. 2024; Haberstroh and Werner 2022; Sachsenmaier et al. 2024; Shovon et al. 2024). This also holds in our study when considering only the neighbourhood species richness of focal trees, without incorporating a functional perspective. Even though, neighbourhood species richness can significantly affect tree growth overall, its effect was not consistently positive during drought conditions (see Figure S13). A recent assessment of functional diversity effects on tree growth responses to drought across Europe did not identify clear positive diversity effects but instead a strengthening of both positive and negative diversity effects during consecutive drought years (Serrano‐León et al. 2025). Unlike previous studies on the 2018–2020 drought, our study focused on neighbourhood‐level interactions and a broad set of hydrologically relevant traits of a focal tree and their neighbours measured at our study site. Using this approach, we revealed that hydro‐functional dissimilarity was beneficial for growth only during drought conditions. In non‐drought years, when water is not limiting, competition for other resources (such as light or nutrients) may override any advantages of hydro‐functional complementarity or facilitation, leading to no significant growth advantage. Thus, the positive effect likely emerges only when water becomes the primary constraint. We assume that high dissimilarity reduces competition and enhances complementarity in species interactions during drought, though the specific tree‐neighbour interactions behind this effect remain unclear, and we cannot disentangle whether these effects arise from complementarity, facilitation, or a combination of both. Several mechanisms may potentially be involved: When trees keep their stomata open despite drought conditions and continue drawing water from the soil, they benefit from neighbours that close their stomata and conserve soil moisture, reducing direct competition for water (Lübbe et al. 2016). Alternatively, when dominant tree species droop their leaves as a result of drought, formerly suppressed trees may receive more light. While belowground interactions remain unexplored, differences in hydraulic strategies may reflect variations in rooting architecture and depths, potentially enhancing resource partitioning and reducing competition (Mas et al. 2024), especially as woody plants can shift water uptake to deeper soil layers when shallow soils dry out within short timescales (Bachofen et al. 2024). While these mechanisms reflect complementarity, facilitative interactions may also contribute to the positive effects of hydro‐functional dissimilarity. Continued transpiration improves the local microclimate by reducing heat load and atmospheric drought in their immediate surroundings (Richter et al. 2022), helping neighbours cope with local drought stress and lowering the risk of heat‐related leaf damage. Continued transpiration may also facilitate hydraulic redistribution of water through the root system (vertically and laterally), potentially benefiting neighbouring trees with limited water access (Hafner et al. 2021; Neumann and Cardon 2012). The only negative relationship between hydro‐functional dissimilarity and growth appeared in 2017 (Figure 4). In 2017, the onset of canopy closure may have intensified competition for light to such an extent that it created substantial stress or competitive disadvantages that could not be offset by functional differences. Dissimilarity in hydraulic safety had a slightly stronger positive effect on growth during drought than dissimilarity in stomatal control potential (Figure 4b,c), possibly due to the drought's severity. Hydraulic safety traits may have been more relevant under these conditions, or this axis, including xylem safety and LES traits, may simply capture a wider range of functional variation than stomatal control potential alone.

By combining hydro‐functional identity and dissimilarity effects, we could elucidate which species benefit from dissimilarity under different conditions (Figure 5). For stomatal control potential, dissimilarity offered no benefit in wet years for either high‐ or low‐control species, likely because ample water availability makes stomatal strategies less relevant. During drought, however, both groups benefited from dissimilarity, possibly because high‐control species faced less competition from inactive neighbours, while low‐control species gained from improved microclimates in more diverse neighbourhoods. In contrast, the hydraulic safety identity showed stronger modulation of the dissimilarity effects (Figure 5). During drought, the roles shifted: hydraulically safe species benefited when surrounded by species with low hydraulic safety that shut down, thereby reducing competition; however, among similarly active neighbours, competition increased. For species with lower hydraulic safety, dissimilarity offered no clear benefit. They performed better among similar neighbours in wet years, while during drought, their shutdown strategy may have made neighbour traits less relevant. Our findings contrast with previous studies that found acquisitive and drought‐vulnerable species to profit most from neighbourhood diversity during drought (Fichtner et al. 2020; Schnabel et al. 2024). On the one hand, acquisitive species may have been too severely affected by the drought in our study to benefit from the reduced competition by their less acquisitive neighbours. On the other hand, previous studies focused separately on traits of the focal tree and traits of the neighbourhood (Schnabel et al. 2024) without addressing the hydro‐functional dissimilarity between a tree and its neighbouring trees, making direct comparisons of results challenging. We propose that during severe drought, the trait identities of both the tree and its neighbours are critical, with competition being minimized only when trait differences are maximized.

At first glance, functional diversity and dissimilarity may appear to capture similar aspects of trait variation. However, while common diversity indices, such as functional divergence (FDiv) quantify how traits are spread around a community's trait centroid (Villéger et al. 2008), they do not account for how individual trees relate to their specific neighbours. We tested FDiv as a predictor of growth and found similar patterns of positive effects during drought (Figure S14); yet this index treats all species as equally contributing, including the focal tree. In contrast, hydro‐functional dissimilarity captures how distinct a focal tree's traits are from those of its immediate neighbours, and thus may provide a more sensitive lens into neighbourhood‐level interactions, especially under drought.

Numerous studies highlighted the crucial role of growth strategies in driving drought responses within tree mixtures (Guillemot and Martin‐StPaul 2024; Mas et al. 2023; Paligi et al. 2024). To isolate functional effects, we accounted for competition in our models (see Methods). However, differences in growth rates lead to size differences between trees which still shape key drought‐related processes, such as greater total leaf area in larger trees, increasing transpiration, or smaller root systems in suppressed trees, limiting water uptake (Guillemot and Martin‐StPaul 2024; Paligi et al. 2025). Ultimately, the interplay between water use and growth strategies determines how diverse forests respond to drought. As size ratios shift over time, a hydro‐functional perspective may better capture stable, size‐independent advantages under drought. While the study's basic design could also be applied to natural forests, the experimental setting allowed us to control for confounding factors such as tree age, spatial distance to neighbouring trees, and environmental heterogeneity. Our study focuses on tree growth during an early phase of stand development (6 years after planting), and while juvenile growth trajectories may differ from those of mature trees, the patterns we observed provide valuable insights that could inform reforestation efforts.

## 5 Conclusions

Overall, we can draw the following conclusions from our results: First, hydro‐functional identity plays a crucial role during drought with traits that were less advantageous in normal conditions becoming beneficial under drought stress. Hence, relying on single tree species with specific hydro‐functional strategies suited for either benign or harsh climatic conditions may not be an effective approach. Instead, both groups of hydro‐functional identities are relevant under increasing climatic variability. Second, our results show that hydro‐functional dissimilarity to the surrounding neighbourhood was advantageous during drought and in most cases, not disadvantageous during normal years. Thus, our study contributes to unravelling which water‐use strategy groups benefit under severe drought and draws attention to hydro‐functional dissimilarity as a more relevant indicator of functional diversity at the neighbourhood scale. Acknowledging that further work is needed to translate our findings into specific forest management applications, our results suggest that planting diverse mixtures composed of species with distinct hydro‐functional strategies rather than focusing solely on high species diversity is a suitable silvicultural adaptation strategy to future drought conditions.

## Author Contributions

**Lena Sachsenmaier:** conceptualization, data curation, formal analysis, investigation, methodology, project administration, visualization, writing – original draft, writing – review and editing. **Florian Schnabel:** conceptualization, methodology, supervision, writing – review and editing. **Fon R. Tezeh:** investigation, methodology, writing – review and editing. **Pablo Castro Sánchez‐Bermejo:** investigation, validation, writing – review and editing. **Nico Eisenhauer:** funding acquisition, investigation, resources, writing – review and editing. **Olga Ferlian:** investigation, resources, writing – review and editing. **Sylvia Haider:** investigation, writing – review and editing. **Ronny Richter:** data curation, methodology, validation, writing – review and editing. **Sharath S. Paligi:** investigation, methodology, writing – review and editing. **Bernhard Schuldt:** conceptualization, methodology, resources, validation, writing – review and editing. **Christian Wirth:** conceptualization, funding acquisition, methodology, resources, supervision, validation, writing – review and editing.

## Conflicts of Interest

The authors declare no conflicts of interest.

## Supporting information


**Data S1:** gcb70588‐sup‐0001‐Supinfo.pdf.

## Data Availability

The data that support the findings of this study are openly available in the MyDiv database at the following DOI URLs: tree growth and neighbourhood data at https://doi.org/10.25829/7q2e‐tf69, trait data at https://doi.org/10.25829/4hbe‐ne61 (branch traits), https://doi.org/10.25829/1X09‐RE46 (stomata traits), https://doi.org/10.25829/MCFB‐QD81 (stomatal conductance traits), https://doi.org/10.25829/Z5M9‐4T33 (leaf δ13C), https://doi.org/10.25829/SFH0‐GA09 (water potential data). SLA and LDMC data were obtained from a previously published dataset by Castro Sánchez‐Bermejo et al. (2024) via Zenodo (https://doi.org/10.5281/zenodo.10654726); only the monoculture plots were used in this study. Species means of all 14 hydro‐functional traits are available at https://doi.org/10.25829/505w‐7z44. The R code that supports the findings of this study is openly available in the MyDiv database at https://doi.org/10.25829/pm8z‐7622.
