# Supplementary material for: Hydro‐Functional Traits and Their Dissimilarity to the Neighbourhood Buffer Tree Growth Against the 2018–2020 Central European Drought
Source: Glob Chang Biol. 2025 Nov 13;31(11):e70588. doi: 10.1111/gcb.70588 (PMC12612798; doi:10.1111/gcb.70588)
Supplement: Supplementary file 1 — Data S1: gcb70588‐sup‐0001‐Supinfo.pdf. [file GCB-31-e70588-s001.pdf]

Supplementary Material

Hydro-functional traits and their dissimilarity to the neighbourhood buffer tree growth against the 2018–2020 Central European drought

Lena Sachsenmaier, Florian Schnabel, Fon R. Tezeh, Pablo Castro Sánchez-Bermejo,

Nico Eisenhauer, Olga Ferlian, Sylvia Haider, Ronny Richter, Sharath S. Paligi,

Bernhard Schuldt, Christian Wirth

Contents

Supplementary Material (additional text passages) 2

S1 – Data cleaning procedure 2

S2 – Detailed description of the measurements of functional traits 2

S3 – Discussion of the trait coordination 8

Supplementary Tables 10

Supplementary Figures 21

References 32

Supplementary Material (additional text passages)

S1 – Data cleaning procedure

Measurement errors are common in annual tree inventory data due to inconsistent measurement positions, selection of the main stem in multi-stemmed trees, or stem breakage and regrowth. To identify errors, an error distance was calculated for each diameter measurement, based on the difference between the observed value and the mean of the preceding and following year, normalized by the observed diameter:

$$error\_distance_{year_x} = \left| \frac{mean(diameter_{year_{x-1}}, diameter_{year_{x+1}}) - diameter_{year_x}}{diameter_{year_x}} \right| \quad (1)$$

Measurements with an error distance >0.5 or showing negative growth were flagged for correction. Corrections were performed using two linear regression models per individual tree: one predicting ground-level diameter (d0) from DBH and another from tree height. The DBH model was preferred when a complete DBH series existed; otherwise, the height model was used. Models with $R^2 < 0.5$ were considered unreliable, and original values were retained instead. The cleaning procedure was performed in three consecutive rounds to prevent errors from only shifting to adjacent years. Ultimately, 819 diameter values (5.23%) were corrected. For further details on the procedure and examples, please see Supplementary material of Sachsenmaier et al. (2024).

S2 – Detailed description of the measurements of functional traits

Embolism resistance

For the determination of tree species' embolism resistance, we sampled branches of randomly chosen tree individuals within each monoculture plot of the experiment. Each sample consisted of one straight, healthy, and sun-exposed canopy branch per tree individual. For diffuse-porous species (Tab. S2), we took branches of approximately 60 cm length and 0.3 – 1.0 cm diameter from nine to twelve tree individuals per species (four to six per monoculture) in November 2021. We cut off all side branches with approximately a 1-2 mm distance to the main branch and kept the samples dark, moist, and cool until the measurements in the laboratory began. For cavitation resistance measurements, we cut each sampled branch into a segment of 27.5 cm length and stripped 5 cm of bark off from the upper and the lower ends. The branch segments were placed into a specially designed Cavitron rotor chamber (Cochard et al., 2005) connected to a centrifuge (Sorval RC-5C Plus; Thermo Fisher Scientific) with manual control of rotation speed. The measurements were taken with the CaviSoft software (CaviSoft 4.0, University of Bordeaux, France). We began at pressures of -0.834 MPa and gradually increased the

xylem water pressure via rotation speed until the percentage loss of conductivity (PLC) reached a minimum of 90%. Vulnerability curves were fitted in R version 4.1.2 (R Core Team, 2021) using non-linear least squares with a modified logistics model (Pammenter & van der Willigen, 1998), adapted for hydraulic conductivity (Ogle et al., 2009):

$$K_i \approx Normal\left(K_{sat} \times \left(1 - \frac{1}{1 + \exp\left(-\frac{S_{50}}{25} \times (P_i - P_{50})\right)}\right), \sigma\right) \quad (2)$$

where for each observation *i*, the conductivity K_i is assumed to be normally distributed with residual standard deviation σ around a logistic function of the water potential P_i with the parameters P_{50} (water potential at 50% loss of conductivity), S_{50} (corresponding slope of the vulnerability curve on the percentage loss of conductivity scale) and K_{sat} (conductivity at full saturation).

For the two ring-porous species (*Q. petraea* and *F. excelsior*), we could not use the same methodological approach as for the other species due to their characteristically long vessel elements, which would be compromised if branches were cut into shorter segments of 27.5 cm. To avoid open vessel ends creating measurement artifacts (Cochard et al., 2010; Schuldt et al., 2025), we collected sun-exposed long branches (~150 cm) from four tree individuals per species (two per monoculture) in August 2024. Branches were immediately recut under water to relax tension in the xylem and transported to the laboratory while being placed in water and covered by opaque plastic bags to limit transpiration. The samples were stored in the dark under cool conditions at 8°C until measurements in the laboratory started. For measuring the xylem vulnerability of ring-porous species, we used the optical method at the leaf level (Brodribb et al., 2016; Gauthey et al., 2020) with custom-built camera mounts for the Raspberry Pi camera, capturing images at 30x magnification, called *cavicams* (cavicam.co, Hobart, TAS, Australia). Throughout the drying process, images were taken every five minutes (on one leaf per branch) and analyzed using the Fiji ImageJ software (Fiji Team, 2024, Fiji: ImageJ; as described in http://www.opensourceov.org/process/). The leaf water potentials of adjacent leaves on the same branch were measured using a *Scholander* pressure chamber (Model 1505D, PMS instruments, Albany, OR, USA) initially circa every 1-3 h (with sampling gaps overnight between 23 h and 8 h, however, the water potential difference between previous value was not more than -1.0 MPa). The relationship of water potential over elapsed time was modelled using shape constrained additive models. Monotone increasing P-splines (number of basic functions k = 8) were fitted using the s() function from the R-package *mgcv* (Wood, 2025) within the *scam()* function of the R-package *scam* (Pya, 2025). The large number of knots was chosen to ensure precise fits to the observed water potentials. Overfitting issues were considered less important as the model predictions were only used for the respective curve and not for new data of other branches or individuals. Similar to the centrifuge-based estimation, we determined the P_{50} as the

xylem water potential at which 50% of the xylem area was embolised. Due to unreliable measurements caused by methodological artifacts at our study site for *F. excelsior*, we instead utilized P_{50} estimates from an ongoing study in the nearby Hainich Forest (51°04'46''N, 10°27'08''E). In that study, a similar protocol for sampling, analysis, and optical-method–based P_{50} estimation was employed, ensuring methodological consistency.

Vessel Size, Vessel Density and Wood Density

For xylem anatomical traits, thin wood cross sections (20 µm thick) were prepared from ca. 3-cm long segments from the basipetal end of each sampled branch, using a sliding microtome (G.S.L.1; Schenkung Dapples, Zürich, Switzerland). The sections were stained with safranin-alcian blue, rinsed with distilled water and 99.8% ethanol, and permanently mounted on glass slides with Euparal (Carl Roth, Karlsruhe, Germany). The oven-dried slides (50°C for 10 days) were then digitized at 100x magnification using a stereo-microscope with an automated stage and a digital camera (Observer.Z1, Carl Zeiss Micro Imaging GmbH, Jena, Germany). Anatomical measurements were conducted through semi-automated image analysis with ImageJ (v1.53k) and GIMP (v2.10.34), using the particle analysis function. We estimated vessel density (VD, n mm^{-2}) and calculated the vessel diameter (D, µm) following White (1991), accounting for elliptical vessels with minor and major radii a and b:

$$D = \left(\frac{(32\ (a \times b)^3)}{(a^2 + b^2)}\right)^{\frac{1}{4}} \quad (3).$$

Since, according to the Hagen-Poiseuille law, the volume flow rate through a cylindrical vessel is proportional to the fourth power of the vessel's radius, we calculated the hydraulically weighted average vessel diameter (Dh, µm) (Sperry et al., 1994) as:

$$D_h = \frac{\sum D^5}{\sum D^4} \quad (4).$$

Wood density (WD, g cm^{-3}) was calculated as the ratio of dry mass to fresh volume for ca. 5 cm pieces cut from the original branch samples. After removing the pith and bark from these sections, the fresh volume was measured using water displacement, following Archimedes' principle. The samples were then oven-dried at 105°C for 72 h and weighed to determine their dry mass.

Stomata Density and Size

In July 2022, we sampled three leaves from five randomly selected tree individuals per monoculture plot. We ensured that they were healthy, mature, and sun-exposed leaves from the top canopy. Leaves were stored cool and moist until further processing. In the laboratory, we created leaf imprints from the abaxial surface (at the widest part of the leaf blade, avoiding major veins), using transparent nail polish and adhesive tape. These imprints were transferred onto objective slides and examined under a standard

light microscope (Leica, DM300). Stomatal density was assessed by counting stomata at three different positions per imprint at 400x magnification and calculated in stomata per mm^2 based on the microscope's field of view area. For stomata size analysis, we measured guard cell length at 1000x magnification, using the scale in the microscope's ocular lens.

Leaf Water Potentials and Hydroscape Area

In August 2022, during an exceptionally dry summer period at our study site (Fig. S5), leaf water potentials were assessed at both predawn (ca. 3:00 - 5:00 AM) and midday (ca. 12:00 - 02:00 PM). We randomly selected three trees per monoculture, totalling six individuals per species. To control for environmental variability, one individual from each species was measured on the same day. For each selected tree, three fully expanded, healthy and sun-exposed leaves from the upper canopy were chosen for both predawn and midday measurements. These leaves were cut individually and transported to the measurement area within a maximum of 90 seconds to minimize the time between sampling and assessment. Upon arrival, each leaf's petiole was trimmed to ensure a clean cut. We measured leaf water potentials using a *Scholander* pressure chamber (Scholander et al., 1965; PMS Instrument Company, Model 1505D). We used the midday leaf water potentials (Ψ_{MD}) during drought as a proxy for minimum leaf water potential (Ψ_{min}) and the delta between midday and predawn water potentials ($\Delta\Psi$) was calculated to assess water loss throughout the day.

To assess a species' degree of isohydry, we estimated hydroscape areas (HSA), following the approach outlined by (Meinzer et al., 2016). In brief, HSA was calculated as the area of the triangle formed by the regression line between predawn (Ψ_{PD}) and midday (Ψ_{MD}) leaf water potentials and the 1:1 line (see Fig. S12). Specifically,

$$HSA = \frac{a \times b}{2} \quad (5),$$

where *a* represents the intercept of the linear regression between Ψ_{PD} and Ψ_{MD}, reflecting the most negative Ψ_{MD} when Ψ_{PD} equals zero, and *b* is the point where the regression line intersects with the 1:1 line, corresponding to $\Psi_{PD} = \Psi_{MD}$. Since HSA reflects the water potential landscape over which stomata are effective in controlling leaf Ψ, species with larger hydroscapes are expected to be more anisohydric.

Stomatal Conductance

During the same days, we assessed stomatal conductance (g_s) of the same tree individuals. Prior to the measurements, we selected seven fully-expanded, healthy leaves from the upper canopy for each tree individual and labelled them. Using ladders to access the upper canopy, we repeatedly measured all seven leaves of each tree individual during the course of the day in 1 – 2 h intervals, between ca. 6:30 AM and 3:00 PM. Stomatal conductance was measured with a hand-held porometer device (LI-600, LI-COR, Lincoln, Nebraska USA), under fully clear sky conditions to avoid any stomatal reactions due to

changes in irradiation. Due to extreme drought conditions, stomatal conductance was overall low and for many species often in the range of minimal stomatal conductance. Out of all measurement points per species, we calculated the mean stomatal conductance and called it g_s *(drought)*, since it reflects a species´ ability to transpire despite drought conditions. Additionally, we approximated g_{res} using all values of stomatal conductance that were measured at a leaf vapor pressure deficit (VPD) higher than 3 kPa, which reflects extreme conditions at which we assume stomata to be closed. We consider the trait g_{res} used in this study as a field-measured value that approximates minimal conductance; however, this approach should not be directly compared to ex situ lab measurements of g_{min}, as it does not account for instances where minimal conductance may not be achieved due to leaf cooling, leading to values remaining above the actual minimum of a certain species (Machado et al., 2021).

Leaf Carbon Isotopes

In September 2022 (end of growing season), we sampled leaves on three randomly selected tree individuals in each of the monoculture plots (n = 6 trees per species). From each tree, five healthy, fully expanded, sun-exposed leaves from different parts of the upper canopy were collected and pooled into one sample. Leaves were dried immediately after collection at 60°C for 48 h and subsequently shredded (cutting mill SM 100, RETSCH) and milled (mixer mill MM 400, RETSCH, Haan, Germany) to obtain a homogenous leaf powder sample per tree individual. Of each sample, we weighed 0.8 mg (± 0.015 mg) into tin capsules. Carbon isotope measurements were performed using an elemental analyser (NA1110, CE Instruments, Milan, Italy) connected to a Delta+XL isotope ratio mass spectrometer (Thermo Finnigan, Bremen, Germany) *via* a ConFlow III interface. These analyses were carried out at the BGC-IsoLab, the sTab. isotope facility of the Max Planck Institute for Biogeochemistry in Jena, Germany. Leaf carbon isotope discrimination ($\delta^{13}C$) values were calculated as the relative difference between the $^{13}C/^{12}C$ ratio of the sample and that of the international standard Vienna PeeDee Belemnite (VPDB) and expressed in per mil (‰), following Farquhar et al. (1989):

$$\delta^{13}C = \frac{\delta^{13}C\ (sample)}{\delta^{13}C\ (standard)} - 1 \quad (6).$$

Samples were calibrated against an in-house standard (acetanilide) with a $\delta^{13}C$ value of −30.06 ± 0.1‰. As a quality control, caffeine (caf-j3; $\delta^{13}C$ = −40.46 ± 0.1‰) was analysed repeatedly within each measurement run. To ensure accuracy and consistency over time, in-house standards at BGC-IsoLab are cross-checked biannually against internationally recognized standards IAEA-603 (+1.46 ± 0.01‰) and NBS22 (−30.03 ± 0.04‰), following recommendations by Dunn et al. (2021). Each analytical sequence included corrections for linearity, blanks, and instrument drift (Werner & Brand, 2001).

Specific Leaf Area and Leaf Dry Matter Content

In August 2021, we sampled fully developed, healthy leaves from 16 tree individuals per species in monoculture plots, collecting five leaves per tree at different heights along the canopy. The leaves were

analysed using visible-near infrared reflectance spectrometry (Vis-NIRS). Specific leaf area (SLA; leaf area divided by leaf dry mass) and leaf dry matter content (LDMC; leaf dry mass divided by leaf fresh mass) were predicted using a convolutional neural network approach (Vasseur et al., 2022), trained on a calibration set of leaves measured with standard laboratory protocols. The quality of the predictive models was addressed by assessing the coefficient of determination (R^2) for the predicted and measured values in an independent set of samples for validation. R^2 were 0.88 for SLA and 0.86 for LDMC. For further details on sampling and analytical procedures, see Castro Sánchez-Bermejo (2024).

S3 – Discussion of the trait coordination

While it is widely recognised, both theoretically and empirically, that stomatal closure decreases transpiration, thereby reducing the water potential gradient between the soil and leaves and lowering the risk of hydraulic failure (Cochard, 2002; Fu & Meinzer, 2019; Klein, 2014; McDowell et al., 2008; Sperry et al., 2002), this might only be true within a tree, but not across species (Martínez-Vilalta & Garcia-Forner, 2017). Our results point to the option that stomatal control and hydraulic safety operate more independently than previously thought, maybe through different temporal dimensions: stomatal regulation may be most critical from the onset of drought until stomatal closure, whereas hydraulic safety becomes the dominant factor afterwards (Blackman et al., 2016; Martin-StPaul et al., 2017; Waite et al., 2024). Similar results of two orthogonal gradients of stomatal control and cavitation resistance have been observed for tree species of the subtropics (Kröber et al., 2014; Schnabel et al., 2024) and also the temperate zone (Waite et al., 2024). However, in our study, the two traits water potential differences between midday and predawn ($\Delta\Psi$) and hydroscape area (HSA) were associated with the gradient of hydraulic safety. Both are expected to be inherently linked to xylem resistance but also to stomatal regulation - often referred to as key indicators of the degree of isohydry (Fu & Meinzer, 2019; Meinzer et al., 2016). Therefore, we expected, in particular, HSA to be relevant to both PCA axes or, as in other studies, to be more closely associated with stomatal stringency (Waite et al., 2024). However, the pattern we found may be explained by the exceptional drought conditions during the measurements, suggesting that when stomatal regulation reaches its limits, the prevention of large fluctuations in water potential (high $\Delta\Psi$ and HSA) depends more on xylem traits. Importantly, the exclusion of $\Delta\Psi$ and HSA did not change the orthogonality of hydraulic safety and stomatal control in our study (see the PCA without isohydry traits in Supplementary Fig. S8). Although xylem anatomical traits were expected to align with other hydraulic safety traits like P_{50} (Avila et al., 2023; Isasa et al., 2023), they instead formed a third axis of variation (Fig. S5), reflecting the well-established trade-off between vessel density and size (Zanne et al., 2010). However, this axis did not add any explanatory power beyond the first two and was not used in further analysis.

On the hydraulic safety gradient, species with a high $\Delta\Psi$ and HSA were associated with rather conservative strategies, possibly delaying stomatal closure and relying on either high xylem resistance (Brodribb et al., 2003; Klein, 2014) or on belowground systems like a more extended root system or higher fine root acclimation potential (Jaeger et al., 2024; Kahmen et al., 2022). In contrast, acquisitive species may depend more on greater water capacitance in their tissue, as indicated by their lower wood density (Ziemińska et al., 2020). As expected, embolism resistance correlated negatively with leaf minimum water potential, suggesting that species tolerant to low water potentials before substantial embolism can also risk sharp midday water potential drops during drought (Martínez-Vilalta & Garcia-Forner, 2017). The summer drought of 2022, during which we measured the water potentials, had a similar severity compared to the drought years 2019 and 2020 (see Fig. S2). This is why we have good

reason to assume that embolisms should also have occurred in 2019 and 2020. This is even more likely for the more extreme year 2018. We found SLA, LDMC, and wood density to align with the hydraulic safety axis (**Fig. 2**). This suggests that species with more conservative resource-use strategies - characterised by robust, long-lived leaves (high LDMC) and denser wood - also possess traits that enhance hydraulic safety (Guillemot et al., 2022; Reich, 2014). Previous studies have also demonstrated that high embolism resistance is closely linked to the conservative spectrum of leaf and xylem traits (Da Sois et al., 2024; Eller et al., 2018; Fan et al., 2011; Kröber et al., 2014; Maherali et al., 2006; Oliveira et al., 2021; Schumann et al., 2019). Further, thicker and tougher leaves were also found to have a higher thermal tolerance (Münchinger et al., 2023), which can be an important advantage during heat waves of summer droughts, like in 2018.

Our finding that species with high mean stomatal conductance during drought also displayed high residual conductance under extreme vapour pressure deficits may be explained by two not mutually exclusive mechanisms: either species with many small stomata regulated them with high precision to enable cooling through transpiration, or increased stomatal density led to more frequent leakage or water loss due to incomplete stomatal closure under extreme conditions (Brodribb et al., 2014; Duursma et al., 2019; Machado et al., 2021). While previous studies have linked smaller stomata to higher intrinsic water-use efficiency and increased $\delta^{13}C$ under normal conditions (Petrík et al., 2024), we found that stomatal size was associated with higher $\delta^{13}C$, during drought conditions, possibly due to species with larger stomata having less precise regulating options, avoiding hydraulic failure by keeping them mostly closed (Cernusak et al., 2013). The existence of universal trait syndromes that define forest responses to drought remains a debated topic (Leuschner et al., 2019; Martínez-Vilalta & Garcia-Forner, 2017; Oliveira et al., 2021; Waite et al., 2024) and requires further research, particularly regarding stomatal control traits for which we still lack a unified definition of their quantification.

With our trait selection, we could cover important aspects of water-use strategies and drought tolerance, but we miss the belowground part that is particularly critical for drought responses (Baca Cabrera et al., 2024; Comas et al., 2013), such as mycorrhizal colonisation (Lehto & Zwiazek, 2011; Lv et al., 2023) , rooting depth (Kahmen et al., 2022) or root shrinkage potential (Duddek et al., 2022; Nye, 1994). Nevertheless, our study is among the few that include a comprehensive set of 14 hydro-functional traits, all measured consistently at the same site, at trees of the same age, and with the same methods, providing a solid basis for analysing hydro-functional strategies.

Supplementary Tables

Tab. S 1: Hydro-functional traits used in this study, including their units, definitions, and contributions (%) to the first two principal components (PC1 and PC2) of the PCA. Contribution values greater than the expected average (1/number of traits = 7.1%) are shown in black; contribution values below average are shown in grey.

**Trait**	**Unit**	**Description**	**Contribution to PC1 [%]**	**Contribution to PC2 [%]**
P50	MPa	Xylem water potential at a 50 percent loss of hydraulic conductance	12.86	0.53
Ψ_{min}	MPa	Minimum leaf water potential calculated as the midday water potential during summer drought	14.02	2.21
$\Delta\Psi$	MPa	Delta leaf water potential calculated as the difference between the midday and the predawn leaf water potential during summer drought	16.09	1.22
HSA	MPa^2	Hydroscape area, calculated as the area of the triangle formed between the 1:1 line and the regression line made between $\Psi_{predawn}$ and Ψ_{midday}	16.45	0.14
WD	g cm^{-3}	Branch Wood Density	8.03	7.91
SLA	mm^2 mg^{-1}	Specific Leaf Area, calculated as leaf area divided by its dry mass	7.75	1.40
LDMC	mg g^{-1}	Leaf Dry Matter Content	8.36	5.11
SD	N mm^{-2}	Stomatal density	3.08	10.34
GCL	µm	Guard cell length	0.01	18.88
gs_drought	mmol m^{-2} s^{-1}	Stomatal conductance, calculated as the mean amount of water lost per area and per time measured from morning till afternoon during a summer drought period	5.53	14.29
g_res	mmol m^{-2} s^{-1}	Leaf residual conductance, calculated as the amount of water lost per area and per time when leaf vapor pressure deficit was > 3 kPa	4.42	20.46
$\delta^{13}C$	‰	Leaf carbon isotope discrimination	0.05	16.89
VD	N mm^{-2}	Branch Vessel Density	3.18	0.59
D_h	µm	Branch Vessel Diameter, hydraulically weighted	0.17	0.029

Tab. S 2: ***Characteristics of tree species included in the study.*** *For each species, the table provides the species code, taxonomic family, total number of trees included, mean tree height in 2021 (m), cumulative mortality until 2021 (%), and wood porosity type (from the TRY database, Kattge et al., 2020). The number of trees is further detailed by plot species richness (monocultures, two-species mixtures, and four-species mixtures).*

**Species code**	**Species**	**Family**	**Plot species richness**	**Nr. of trees included in the study**	**Mean height in 2021 [m]**	**Mortality [%] (until 2021)**	**Wood porosity (TRY Database; (Kattge et al., 2020)**
**Ac**	*Acer pseudoplatanus* L.	Sapindaceae	total	275	5.6	4.7	diffuse
			1	65			
			2	103			
			4	107			
**Ae**	*Aesculus hippocastanum* L.	Sapindaceae	total	278	3.2	2.9	diffuse
			1	64			
			2	107			
			4	107			
**Be**	*Betula pendula* Roth	Betulaceae	total	208	6.0	21.9	diffuse
			1	33			
			2	84			
			4	91			
**Ca**	*Carpinus betulus* L.	Betulaceae	total	282	4.6	1.2	diffuse
			1	71			
			2	106			
			4	105			
**Fa**	*Fagus sylvatica* L.	Fagaceae	total	245	2.6	12.7	diffuse
			1	71			
			2	94			
			4	80			
**Fr**	*Fraxinus excelsior* L.	Oleaceae	total	260	4.8	10.7	ring
			1	60			
			2	99			
			4	101			
**Pr**	*Prunus avium* (L.) L.	Rosaceae	total	284	5.7	1.4	semi-ring
			1	70			
			2	106			
			4	108			
**Qu**	*Quercus petraea* (Matt.) Liebl.	Fagaceae	total	249	2.8	12.1	ring
			1	67			
			2	93			
			4	89			
**So**	*Sorbus aucuparia* L.	Rosaceae	total	250	4.5	13.5	diffuse
			1	71			
			2	98			
			4	81			
**Ti**	*Tilia platyphyllos* Scop.	Malvaceae	total	280	4.5	2.0	diffuse
			1	67			
			2	106			
			4	107			

Tab. S 3: ***Species compositions in the MyDiv experimental plots across richness levels.*** *The experiment consists of 20 monocultures, 30 2-species mixtures, and 30 4-species mixtures (total 80 plots), distributed across two blocks. Cells show the compositions of the plots with bold font indicating compositions that are replicated across both experimental blocks (species codes: Ac = Acer pseudoplatanus, Ae = Aesculus hippocastanum, Be = Betula pendula, Ca = Carpinus betulus, Fa = Fagus sylvatica, Fr = Fraxinus excelsior, Pr = Prunus avium, Qu = Quercus petraea, So = Sorbus aucuparia, Ti = Tilia platyphyllos).*

monocultures	2-spp-mix	4-spp-mix
**Ac**	Ac|Ae	Ac|Ae|Be|Ca
**Ae**	Ac|Be	**Ac|Ae|Fr|Pr**
**Be**	Ac|Fr	**Ac|Ae|Fr|So**
**Ca**	Ac|Pr	**Ac|Ae|Pr|So**
**Fa**	Ac|So	Ac|Fr|Be|Ti
**Fr**	Ac|Ti	**Ac|Fr|Pr|So**
**Pr**	Ae|Fa	Ac|Pr|Fa|Ti
**Qu**	Ae|Fr	Ac|So|Ca|Qu
**So**	Ae|Pr	**Ae|Fr|Pr|So**
**Ti**	Ae|Qu	Ae|Fr|Qu|Ti
	Ae|So	Ae|Pr|Ca|Ti
	Be|Ca	Ae|So|Be|Fa
	Be|Fa	**Be|Ca|Fa|Qu**
	Be|Qu	**Be|Ca|Fa|Ti**
	Be|Ti	**Be|Ca|Qu|Ti**
	Ca|Fa	**Be|Fa|Qu|Ti**
	Ca|Qu	**Ca|Fa|Qu|Ti**
	Ca|Ti	Fr|Pr|Fa|Qu
	Fa|Qu	Fr|So|Ca|Fa
	Fa|Ti	Pr|So|Be|Qu
	Fr|Ca	
	Fr|Fa	
	Fr|Pr	
	Fr|So	
	Pr|Be	
	Pr|So	
	Pr|Ti	
	Qu|Ti	
	So|Ca	
	So|Qu	

Tab. S 4: Hydro-functional traits for 10 tree species. *Mean (+-standard deviation) values of 14 traits, including water potential at 50 % loss of conductivity (P50), minimum water potential (Ψmin), difference between midday and predawn water potential (ΔΨ), wood density (WD), specific leaf area (SLA), leaf dry matter content (LDMC), stomata density (SD), guard cell length (GCL), mean stomatal conductance during drought (gs_drought), residual conductance at leaf VPD > 3 (g_res), leaf carbon isotope composition (leaf δ13C), hydraulically weighted vessel diameter (Dh), and vessel density (VD).*

	Acer pseudoplatanus	***Aesculus hippocastanum***	***Betula pendula***	***Carpinus betulus***	***Fagus sylvatica***	***Fraxinus excelsior***	***Prunus avium***	***Quercus petraea***	***Sorbus aucuparia***	***Tilia platyphyllos***
P50 [MPa]	-3.57<br>(± 0.3)	-2.33<br>(± 0.2)	-2.19<br>(± 0.1)	-4.41<br>(± 0.4)	-3.19<br>(± 0.4)	-4,24<br>(± 0.8)	-3.9<br>(± 0.4)	-5.11<br>(± 0.9)	-5.16<br>(± 0.4)	-3.11<br>(± 0.4)
Ψmin [MPa]	-0.47<br>(± 0.2)	-0.75<br>(± 0.4)	-1.85<br>(± 0.5)	-2.88<br>(± 0.2)	-2.53<br>(± 0.2)	-3.63<br>(± 1.3)	-3.19<br>(± 0.3)	-2.76<br>(± 0.7)	-3.09<br>(± 0.3)	-1.96<br>(± 0.3)
ΔΨ [MPa]	0.21<br>(± 0.2)	0.31<br>(± 0.2)	0.73<br>(± 0.3)	0.82<br>(± 0.5)	1.33<br>(± 0.3)	1.12<br>(± 0.7)	1.2<br>(± 0.4)	1.36<br>(± 0.3)	1.38<br>(± 0.4)	0.82<br>(± 0.5)
HSA [MPa²]	0.11	0.3	1.65	3.95	3.3	6.47	4.78	5.72	4.76	1.88
WD [g cm⁻³]	0.49<br>(± 0.03)	0.41<br>(± 0.05)	0.52<br>(± 0.04)	0.57<br>(± 0.05)	0.57<br>(± 0.04)	0.5<br>(± 0.06)	0.51<br>(± 0.06)	0.57<br>(± 0.03)	0.52<br>(± 0.06)	0.49<br>(± 0.12)
SLA [mm² mg⁻¹]	16.51<br>(± 2.7)	19.21<br>(± 3.4)	13.62<br>(± 2.1)	18.92<br>(± 3.1)	17.2<br>(± 3.7)	13.83<br>(± 1.9)	14.65<br>(± 2.7)	13.21<br>(± 1.6)	9.61<br>(± 1.6)	21.21<br>(± 2.1)
LDMC [mg g⁻¹]	0.34<br>(± 0.02)	0.32<br>(± 0.03)	0.40<br>(± 0.03)	0.40<br>(± 0.02)	0.42<br>(± 0.02)	0.33<br>(± 0.02)	0.39<br>(± 0.03)	0.40<br>(± 0.02)	0.44<br>(± 0.02)	0.32<br>(± 0.02)
SD [N mm⁻²]	59.94<br>(± 33)	428.05<br>(± 142)	158.38<br>(± 42)	272.67<br>(± 66)	222.65<br>(± 51)	305.23<br>(± 60)	317.31<br>(± 98)	553.59<br>(± 78)	143.29<br>(± 31)	237.74<br>(± 78)
GCL [µm]	20.19<br>(± 4.4)	19.42<br>(± 2.8)	28.94<br>(± 6.5)	22.01<br>(± 3.6)	22.82<br>(± 3.4)	24.62<br>(± 4.8)	20.6<br>(± 5.4)	22.16<br>(± 4.5)	21.64<br>(± 4.9)	24.1<br>(± 4.8)
gs_drought [mmol m⁻² s⁻¹]	11.86<br>(± 9.9)	33.42<br>(± 28.4)	6.01<br>(± 7.8)	25.2<br>(± 43)	28.86<br>(± 40.7)	20.12<br>(± 19.3)	12.78<br>(± 8.5)	40.66<br>(± 26)	25.62<br>(± 14.3)	10.07<br>(± 10.6)
g_res [mmol m⁻² s⁻¹]	9.36<br>(±6.83)	24.59<br>(±13.00)	2.30<br>(±2.45)	13.54<br>(±10.33)	15.69<br>(±10.88)	14.29<br>(±14.69)	9.88<br>(±6.98)	26.19<br>(±24.42)	18.98<br>(±8.06)	6.69<br>(±5.83)
leaf_d13C [‰]	-28.4<br>(± 0.9)	-28.45<br>(± 0.9)	-26.62<br>(± 1.0)	-27.02<br>(± 1.1)	-27.97<br>(± 0.4)	-28.58<br>(± 1.1)	-27.98<br>(± 0.5)	-27.8<br>(± 0.7)	-28.2<br>(± 0.7)	-27.74<br>(± 1.0)
Dh [µm]	42.32<br>(± 3.3)	33.07<br>(± 3.5)	35.97<br>(± 2.8)	40.72<br>(± 3.1)	36.02<br>(± 4.7)	24.46<br>(± 1.5)	31.86<br>(± 3.5)	30.11<br>(± 1.5)	24.92<br>(± 1.6)	23.83<br>(± 5.7)
VD [N mm⁻²]	142.86<br>(± 26)	276.45<br>(± 87)	237.22<br>(± 84)	162.2<br>(± 31)	218.17<br>(± 51)	276.49<br>(± 36)	309.44<br>(± 126)	339.33<br>(± 63)	463<br>(± 120)	1091.92<br>(± 106)

Tab. S 5: *Pairwise functional distances calculated from 14 hydro-functional traits among 10 tree species. Colour coding highlights functional dissimilarity, with red indicating highly similar species and green indicating highly dissimilar species.*

species	*Acer pseudoplatanus*	*Aesculus hippocastanum*	*Betula pendula*	*Carpinus betulus*	*Fagus sylvatica*	*Fraxinus excelsior*	*Prunus avium*	*Quercus petraea*	*Sorbus aucuparia*	*Tilia platyphyllos*
Acer pseudoplatanus	0.000	0.304	0.389	0.380	0.389	0.425	0.345	0.594	0.515	0.379
Aesculus hippocastanum	0.304	0.000	0.513	0.468	0.421	0.459	0.447	0.490	0.560	0.432
Betula pendula	0.389	0.513	0.000	0.341	0.343	0.457	0.343	0.525	0.488	0.363
Carpinus betulus	0.380	0.468	0.341	0.000	0.184	0.335	0.241	0.306	0.331	0.406
Fagus sylvatica	0.389	0.421	0.343	0.184	0.000	0.323	0.219	0.262	0.265	0.381
Fraxinus excelsior	0.425	0.459	0.457	0.335	0.323	0.000	0.202	0.329	0.272	0.343
Prunus avium	0.345	0.447	0.343	0.241	0.219	0.202	0.000	0.268	0.241	0.348
Quercus petraea	0.594	0.490	0.525	0.306	0.262	0.329	0.268	0.000	0.245	0.542
Sorbus aucuparia	0.515	0.560	0.488	0.331	0.265	0.272	0.241	0.245	0.000	0.469
Tilia platyphyllos	0.379	0.432	0.363	0.406	0.381	0.343	0.348	0.542	0.469	0.000

Tab. S 6: *Pairwise functional distances calculated from PC axis scores representing hydraulic safety traits (PC1) and stomatal control traits (PC2) among 10 tree species. Colour coding highlights functional dissimilarity, with lighter colours indicating highly similar species and darker colours indicating highly dissimilar species.*

	PC1 | Hydraulic Safety										PC 2 | Stomatal Control									
**species**	*Acer pseudoplatanus*	*Aesculus hippocastanum*	*Betula pendula*	*Carpinus betulus*	*Fagus sylvatica*	*Fraxinus excelsior*	*Prunus avium*	*Quercus petraea*	*Sorbus aucuparia*	*Tilia platyphyllos*	*Acer pseudoplatanus*	*Aesculus hippocastanum*	*Betula pendula*	*Carpinus betulus*	*Fagus sylvatica*	*Fraxinus excelsior*	*Prunus avium*	*Quercus petraea*	*Sorbus aucuparia*	*Tilia platyphyllos*
Acer pseudoplatanus	0.00	0.09	0.22	0.57	0.62	0.71	0.62	1.00	0.90	0.18	0.00	0.45	0.55	0.16	0.12	0.01	0.10	0.14	0.08	0.12
Aesculus hippocastanum	0.09	0.00	0.14	0.48	0.53	0.62	0.54	0.91	0.82	0.09	0.45	0.00	1.00	0.61	0.57	0.46	0.55	0.31	0.53	0.57
Betula pendula	0.22	0.14	0.00	0.34	0.39	0.48	0.40	0.78	0.68	0.05	0.55	1.00	0.00	0.39	0.43	0.54	0.45	0.69	0.47	0.43
Carpinus betulus	0.57	0.48	0.34	0.00	0.05	0.14	0.06	0.43	0.34	0.39	0.16	0.61	0.39	0.00	0.05	0.15	0.06	0.30	0.09	0.04
Fagus sylvatica	0.62	0.53	0.39	0.05	0.00	0.09	0.01	0.38	0.29	0.44	0.12	0.57	0.43	0.05	0.00	0.10	0.01	0.26	0.04	0.01
Fraxinus excelsior	0.71	0.62	0.48	0.14	0.09	0.00	0.09	0.29	0.20	0.53	0.01	0.46	0.54	0.15	0.10	0.00	0.09	0.15	0.06	0.11
Prunus avium	0.62	0.54	0.40	0.06	0.01	0.09	0.00	0.38	0.28	0.44	0.10	0.55	0.45	0.06	0.01	0.09	0.00	0.24	0.03	0.02
Quercus petraea	1.00	0.91	0.78	0.43	0.38	0.29	0.38	0.00	0.10	0.82	0.14	0.31	0.69	0.30	0.26	0.15	0.24	0.00	0.22	0.26
Sorbus aucuparia	0.90	0.82	0.68	0.34	0.29	0.20	0.28	0.10	0.00	0.72	0.08	0.53	0.47	0.09	0.04	0.06	0.03	0.22	0.00	0.05
Tilia platyphyllos	0.18	0.09	0.05	0.39	0.44	0.53	0.44	0.82	0.72	0.00	0.12	0.57	0.43	0.04	0.01	0.11	0.02	0.26	0.05	0.00

Tab. S 7: *Summary of linear mixed-effects models (LMMs) used in this study. For each model, the formula (fixed and random effects), the data subset included, and the corresponding research question are shown. (Abbreviations: PC1 = hydraulic safety axis, PC2 = stomatal control potential axis, FDissim = functional dissimilarity to neighbourhood.)*

**Model**	**Model formula**	**Data set**	**Corresponding question**
**M1a**	*growth* ~<br>PC1 x drought status +<br>tree size + competition index + (1|year) + (1|plot/tree ID)	Full set	Effect of hydraulic safety on tree growth in drought vs. non-drought years
**M1b**	*growth* ~<br>PC2 x drought status +<br>tree size + competition index + (1|year) + (1|plot/tree ID)	Full set	Effect of stomatal control on tree growth in drought vs. non-drought years
**M2a**	*growth* ~<br>PC1 x year +<br>tree size + competition index + (1|plot/tree ID)	Full set	Effect of hydraulic safety on tree growth across years
**M2b**	*growth* ~<br>PC2 x year +<br>tree size + competition index + (1|plot/tree ID)	Full set	Effect of stomatal control on tree growth across years
**M3a**	*growth* ~<br>FDissim x year +<br>tree size + competition index + (1|plot/tree ID)	Subset without trees in monocultures	Effect of functional dissimilarity to the neighbourhood on tree growth across years
**M4a**	*growth* ~<br>$FDissim_{PC1}$ x year +<br>tree size + competition index + (1|plot/tree ID)	Subset without trees in monocultures	Effect of functional dissimilarity to the neighbourhood regarding hydraulic safety on tree growth across years
**M4b**	*growth* ~<br>$FDissim_{PC2}$ x year +<br>tree size + competition index + (1|plot/tree ID)	Subset without trees in monocultures	Effect of functional dissimilarity to the neighbourhood regarding stomatal control on tree growth across years
**M5a**	*growth* ~<br>FDissim x year x PC1 +<br>tree size + competition index + (1|plot/tree ID)	Subset without trees in monocultures	Interactive effect of functional dissimilarity to the neighbourhood with hydraulic safety on tree growth across years
**M5b**	*growth* ~<br>FDissim x year x PC2 +<br>tree size + competition index + (1|plot/tree ID)	Subset without trees in monocultures	Interactive effect of functional dissimilarity to the neighbourhood with stomatal control on tree growth across years

Tab. S 8: Summary of the principal component analysis (PCA) *for 14 functional traits, including loadings, standard deviation, proportion of the variance explained by each component, the cumulative proportion, the unadjusted eigenvalues and the adjusted eigenvalues obtained in a Horn's parallel analysis using the paran function of the paran package in R.*

**Trait/Metric**	**PC1**	**PC2**	**PC3**	**PC4**	**PC5**	**PC6**	**PC7**	**PC8**	**PC9**	**PC10**
Ψmin	1.571	0.602	-0.449	-1.075	0.110	1.157	-0.964	0.546	-1.372	-1.288
P50	1.513	-0.198	0.257	0.162	1.352	0.156	2.158	-0.517	-0.158	-0.069
HSA	-1.298	-0.073	0.656	-1.125	0.240	-1.781	-0.254	-0.122	0.317	-2.453
ΔΨ	-1.281	-0.335	0.644	-0.802	-0.195	0.194	1.472	-0.261	-0.853	-0.098
SLA	1.218	0.492	0.399	1.381	-1.690	-1.322	0.980	0.297	1.129	0.338
LDMC	-0.869	-0.753	-0.983	-1.252	-0.227	1.435	0.685	-1.338	0.533	0.513
Dh	0.850	-0.214	-1.970	-0.781	-0.506	-1.091	-0.210	-0.135	-0.437	-0.497
WD	-0.848	-0.952	-0.689	0.034	-1.175	-0.307	-0.147	1.420	-1.228	1.410
gs_drought	-0.671	1.430	-0.577	0.389	0.234	0.566	0.064	1.025	1.209	0.698
g_res	-0.579	1.698	-0.306	-0.411	0.465	0.673	-0.336	0.372	1.013	-0.214
SD	-0.452	1.226	0.349	1.754	0.997	-0.975	-0.744	-1.362	-1.625	1.354
VD	0.345	0.003	2.238	-0.038	-1.463	1.252	-0.709	-0.278	-0.246	-0.185
leaf_d13C	0.276	-1.421	-0.400	1.542	0.096	0.164	-1.510	-1.444	1.273	-0.048
GCL	0.223	-1.506	0.830	0.220	1.762	-0.122	-0.484	1.798	0.447	0.540
**Standard deviation**	2.277	1.785	1.489	1.133	0.890	0.786	0.576	0.504	0.367	0.000
**Proportion of variance**	0.370	0.228	0.158	0.092	0.057	0.044	0.024	0.018	0.010	0.000
**Cumulative proportion**	0.370	0.598	0.756	0.848	0.904	0.949	0.972	0.990	1.000	1.000
**Unadjusted eigenvalues**	5.183	3.187	2.217	1.284	0.791	0.617	0.332	0.254	0.135	0.000
**Adjusted eigenvalues**	2.412	1.321	0.958	0.548	0.492	0.697	0.700	0.891	0.981	1.000

Tab. S 9: The effect of PC1 on tree growth – model results: *Linear mixed-effect model (LMM) predicting individual tree growth (log-transformed to meet model assumptions) with interactive fixed effects year (year: 2016-2021) and hydraulic safety strategy (PC1 axis) and the additional fixed effects tree size and competition index (both log-transformed and scaled). We used the tree ID nested within plot ID of the experiment as a random effects structure. The table presents a Type III Analysis of Variance, with F-values and p-values calculated based on denominator degrees of freedom estimated using Satterthwaite's method to test the significance of fixed effects and their interactions.*

**Term**	**Sum_of_Squares**	**Mean_Square**	**Numerator_ DF**	**Denominator_ DF**	**F_value**	**p_value**
PC1_hyd_safe	0.1441953	0.1441953	1	2328.5	13.1	3.0854E-04
year	40.5889300	8.1177860	5	13487.2	735.1	<2.00E-16
tree_size	67.2481913	67.2481913	1	3635.6	6089.4	<2.00E-16
competition_index	11.7613823	11.7613823	1	4890.7	1065.0	<2.00E-16
PC1_hyd_safe:year	1.0326798	0.2065360	5	12869.1	18.7	<2.00E-16
**Marginal R^2**	0.5537317					
**Conditional R^2**	0.5918730					

Tab. S 10: The effect of PC2 on tree growth – model results: *Linear mixed-effect model (LMM) predicting individual tree growth (log-transformed to meet model assumptions) with interactive fixed effects year (year: 2016-2021) and hydraulic safety strategy (PC1 axis) and the additional fixed effects tree size and competition index (both log-transformed and scaled). We used the tree ID nested within plot ID of the experiment as a random effects structure. The table presents a Type III Analysis of Variance, with F-values and p-values calculated based on denominator degrees of freedom estimated using Satterthwaite's method to test the significance of fixed effects and their interactions.*

Term	Sum_of_Squares	Mean_Square	Numerator_DF	Denominator_DF	F_value	p_value
PC2_stom_con	0.1239065	0.1239065	1	2013.6	11.3	7.9E-04
year	40.6200304	8.1240061	5	13437.7	740.6	<2.00E-16
tree_size	64.5085710	64.5085710	1	3329.2	5880.6	<2.00E-16
competition_index	11.5088921	11.5088921	1	4858.8	1049.2	<2.00E-16
PC2_stom_con:year	2.4805263	0.4961053	5	12841.9	45.2	<2.00E-16
**Marginal R^2**	0.5580209					
**Conditional R^2**	0.5929334					

Tab. S 11: The effect of Functional Dissimilarity to the Neighbourhood (FDissim) on tree growth in mixtures– model results: *Linear mixed-effect model (LMM) predicting individual tree growth (log-transformed to meet model assumptions) with interactive fixed effects year (year: 2016-2021) and FDissim (scaled) and the additional fixed effects tree size and competition index (both log-transformed and scaled). We used the tree ID nested within plot ID of the experiment as a random effects structure. The table presents a Type III Analysis of Variance, with F-values and p-values calculated based on denominator degrees of freedom estimated using Satterthwaite's method to test the significance of fixed effects and their interactions.*

Term	Sum_of_Squares	Mean_Square	Numerator_DF	Denominator_DF	F_value	p_value
**FDissim**	0.0045	0.0045	1	1030.14	0.41	5.2E-01
**year**	28.5585	5.7117	5	10240.63	522.83	<2.00E-16
**tree_size**	43.0655	43.0655	1	2505.64	3942.07	<2.00E-16
**competition_index**	10.4393	10.4393	1	3551.30	955.58	<2.00E-16
**FDissim:year**	0.9611	0.1922	5	9759.63	17.59	<2.00E-16
**Marginal R^2**	0.5746570					
**Conditional R^2**	0.6118038					

Tab. S 12: The effect of Functional Dissimilarity in Hydraulic Safety to the Neighbourhood (FDissim_pc1) on tree growth in mixtures – model results: *Linear mixed-effect model (LMM) predicting individual tree growth (log-transformed to meet model assumptions) with interactive fixed effects year (year: 2016-2021) and FDiss_pc1 (scaled) and the additional fixed effects tree size and competition index (both log-transformed and scaled). We used the tree ID nested within plot ID of the experiment as a random effects structure. The table presents a Type III Analysis of Variance, with F-values and p-values calculated based on denominator degrees of freedom estimated using Satterthwaite's method to test the significance of fixed effects and their interactions.*

Term	Sum_of_Squares	Mean_Square	Numerator_DF	Denominator_DF	F_value	p_value
**FDissim_pc1**	0.1751676	0.1751676	1	1391.2	16.0	6.6E-05
**year**	30.8300172	6.1660034	5	10198.5	564.3	<2.00E-16
**tree_size**	45.4416869	45.4416869	1	2565.5	4158.6	<2.00E-16
**competition_index**	10.1846270	10.1846270	1	3564.6	932.1	<2.00E-16
**FDissim_pc1:year**	0.7795170	0.1559034	5	9763.5	14.3	6.1E-14
**Marginal R^2**	0.5747581					
**Conditional R^2**	0.6138210					

Tab. S 13: ***The effect of Functional Dissimilarity in Stomatal Control to the Neighbourhood (FDissim_pc2) on tree growth in mixtures – model results:*** *Linear mixed-effect model (LMM) predicting individual tree growth (log-transformed to meet model assumptions) with interactive fixed effects year (year: 2016-2021) and FDissim_pc2 (scaled) and the additional fixed effects tree size and competition index (both log-transformed and scaled). We used the tree ID nested within plot ID of the experiment as a random effects structure. The table presents a Type III Analysis of Variance, with F-values and p-values calculated based on denominator degrees of freedom estimated using Satterthwaite's method to test the significance of fixed effects and their interactions.*

Term	Sum_of_Squares	Mean_Square	Numerator_ DF	Denominator_ DF	F_value	p_value
**FDissim_pc2**	0.0037316	0.003731647	1	1060.7	0.3	5.6E-01
**year**	30.4762439	6.095248777	5	10218.2	559.3	<2.00E-16
**tree_size**	45.6934705	45.69347045	1	2588.3	4192.5	<2.00E-16
**competition_index**	10.4697121	10.46971211	1	3537.0	960.6	<2.00E-16
**FDissim_pc2:year**	1.2150678	0.243013566	5	9759.3	22.3	<2.00E-16
**Marginal R^2**	0.5753213					
**Conditional R^2**	0.6123025					

Tab. S 14: ***The interactive effects of Functional Dissimilarity to the Neighbourhood (FDiss) and Hydraulic Safety on tree growth in mixtures – model results:*** *Linear mixed-effect model (LMM) predicting individual tree growth (log-transformed to meet model assumptions) with interactive fixed effects year (year: 2016-2021), FDiss (scaled) and Hydraulic Safety (PC1 axis) with the additional fixed effects tree size and competition index (both log-transformed and scaled). We used the tree ID nested within plot ID of the experiment as a random effects structure. The table presents a Type III Analysis of Variance, with F-values and p-values calculated based on denominator degrees of freedom estimated using Satterthwaite's method to test the significance of fixed effects and their interactions.*

Term	Sum_of_Squares	Mean_Square	Numerator_ DF	Denominator_ DF	F_value	p_value
**FDiss**	0.0295440	0.02954	1	870.3	2.7	9.9E-02
**year**	30.4576787	6.09154	5	10137.7	563.4	<2.00E-16
**PC1_hyd_safe**	0.0008303	0.00083	1	1469.9	0.1	7.8E-01
**tree_size**	41.3348079	41.33481	1	2538.5	3823.0	<2.00E-16
**competition_index**	10.6436038	10.64360	1	3522.1	984.4	<2.00E-16
**FDiss:year**	0.9483984	0.18968	5	9736.7	17.5	<2.00E-16
**FDiss:PC1_hyd_safe**	0.5773700	0.57737	1	1259.2	53.4	4.8E-13
**year:PC1_hyd_safe**	0.5650311	0.11301	5	9711.8	10.5	5.1E-10
**FDiss:year:PC1_hyd_safe**	0.2778407	0.05557	5	9725.1	5.1	1.0E-04
**Marginal R^2**	0.5794940					
**Conditional R^2**	0.6138858					

Tab. S 15: ***The interactive effects of Functional Dissimilarity to the Neighbourhood (FDissim) and Stomatal Control on tree growth – model results:*** *Linear mixed-effect model (LMM) predicting individual tree growth (log-transformed to meet model assumptions) with interactive fixed effects year (year: 2016-2021), FDiss(scaled) and Stomatal Control (PC2 axis) with the additional fixed effects tree size and competition index (both log-transformed and scaled). We used the tree ID nested within plot ID of the experiment as a random effects structure. The table presents a Type III Analysis of Variance, with F-values and p-values calculated based on denominator degrees of freedom estimated using Satterthwaite's method to test the significance of fixed effects and their interactions.*

Term	Sum_of_Squares	Mean_Square	Numerator_DF	Denominator_DF	F_value	p_value
**FDissim**	0.03235	0.03235	1	763.3	3.0	8.3E-02
**year**	29.50946	5.90189	5	10117.4	548.2	<2.00E-16
**PC2_stom_con**	0.17772	0.17772	1	1318.6	16.5	5.1E-05
**tree_size**	40.68640	40.68640	1	2275.2	3779.3	<2.00E-16
**competition_index**	9.41880	9.41880	1	3641.8	874.9	<2.00E-16
**FDissim:year**	0.45881	0.09176	5	9723.0	8.5	4.6E-08
**FDissim:PC2_stom_con**	0.00000	0.00000	1	1669.6	0.0	9.9E-01
**year:PC2_stom_con**	1.54148	0.30830	5	9703.1	28.6	<2.00E-16
**FDissim:year:PC2_stom_c**	0.19281	0.03856	5	9715.1	3.6	3.1E-03
**Marginal R²**	0.57949					
**Conditional R²**	0.61389					

Tab. S 16: Estimated slopes of functional dissimilarity (FDissim) *across years at low (-1) and high (+1) values of hydraulic safety (**PC1_hyd_safe**). Slopes were obtained using emtrends() from the emmeans package in R, based on the linear mixed effects model with the interaction factors year, PC1_hyd_safe and FDissim. The table presents the estimated slope (FDiss.trend), standard error (SE), degrees of freedom (df) using kenward-roger method, confidence intervals (lower.CL and upper.CL), t-ratio, and p-value. Slopes that are significantly different from 0 are highlighted in bold (p < 0.05).*

year	PC1_hyd_safe	FDiss.trend	SE	df	lower.CL	upper.CL	t.ratio	p.value
2016	-1	-0.0453	0.0273	7801	-0.0987	0.0082	-1.6601	9.69E-02
2017	-1	-0.1302	0.0267	7719	-0.1826	-0.0779	-4.8759	**1.10E-06**
2018	-1	-0.0121	0.0265	7683	-0.0641	0.0399	-0.4571	6.48E-01
2019	-1	-0.0075	0.0265	7680	-0.0595	0.0445	-0.2822	7.78E-01
2020	-1	-0.0468	0.0264	7655	-0.0986	0.0049	-1.7731	7.63E-02
2021	-1	-0.0908	0.0263	7693	-0.1425	-0.0392	-3.4496	**5.64E-04**
2016	1	0.0412	0.0282	4680	-0.0140	0.0965	1.4634	1.43E-01
2017	1	-0.0634	0.0277	4650	-0.1177	-0.0091	-2.2879	**2.22E-02**
2018	1	0.1430	0.0277	4596	0.0887	0.1974	5.1609	**2.56E-07**
2019	1	0.1757	0.0278	4585	0.1212	0.2303	6.3160	**2.94E-10**
2020	1	0.2242	0.0281	4550	0.1691	0.2793	7.9806	**1.83E-15**
2021	1	0.0621	0.0285	4531	0.0063	0.1180	2.1815	**2.92E-02**

*Tab. S 17: **Estimated slopes of functional dissimilarity (FDissim)** across years at low (-1) and high (+1) values of stomatal control (**PC2_stom_con**). Slopes were obtained using emtrends() from the emmeans package in R, based on the linear mixed effects model with the interaction factors year, PC2_stom_con and FDissim. The table presents the estimated slope (FDiss.trend), standard error (SE), degrees of freedom (df) using kenward-roger method, confidence intervals (lower.CL and upper.CL), t-ratio, and p-value. Slopes that are significantly different from 0 are highlighted in bold (p < 0.05).*

year	PC2_stom_con	FDiss.trend	SE	df	lower.CL	upper.CL	t.ratio	p.value
2016	-1	0.0155	0.0268	6948	-0.0370	0.0680	0.5799	5.62E-01
2017	-1	-0.0217	0.0262	6959	-0.0732	0.0297	-0.8286	4.07E-01
2018	-1	0.0444	0.0262	6952	-0.0069	0.0957	1.6966	8.98E-02
2019	-1	0.0551	0.0262	6996	0.0038	0.1064	2.1048	**3.53E-02**
2020	-1	0.0226	0.0263	7053	-0.0291	0.0742	0.8561	3.92E-01
2021	-1	0.0150	0.0264	7095	-0.0368	0.0668	0.5688	5.69E-01
2016	1	0.0068	0.0270	5752	-0.0462	0.0598	0.2513	8.02E-01
2017	1	-0.1075	0.0267	5757	-0.1598	-0.0551	-4.0258	**5.75E-05**
2018	1	0.0469	0.0268	5713	-0.0057	0.0995	1.7482	8.05E-02
2019	1	0.1020	0.0270	5714	0.0491	0.1550	3.7799	**1.58E-04**
2020	1	0.1000	0.0273	5737	0.0466	0.1534	3.6683	**2.46E-04**
2021	1	-0.0182	0.0277	5760	-0.0725	0.0361	-0.6564	5.12E-01

*Tab. S 18: **Pairwise comparisons of the estimated slopes of functional dissimilarity (FDissim)** at different levels (low = -1; high = 1) **of hydraulic safety** (PC1_hyd_safe) **and stomatal control** (PC2_stom_con) across years. Single slopes were first estimated using emtrends() from the emmeans package in R, and pairwise contrasts were computed using contrast() with the simple = "PC1_hyd_safe" argument. The table presents the estimated contrast (Estimate), standard error (SE), degrees of freedom (df), confidence intervals (lower.CL and upper.CL), t-ratio, and p-value. Significant contrasts (p < 0.05) are highlighted in bold, indicating years where the effect of FDissim differed significantly between high and low hydraulic safety or stomatal control conditions.*

contrast	year	estimate	SE	df	lower.CL	upper.CL	t.ratio	p.value
(PC1_hyd_safe-1) - PC1_hyd_safe1	2016	-0.0865	0.0365	8077	-0.1581	-0.0150	-2.3702	**1.78E-02**
(PC1_hyd_safe-1) - PC1_hyd_safe1	2017	-0.0668	0.0360	7917	-0.1374	0.0038	-1.8558	6.35E-02
(PC1_hyd_safe-1) - PC1_hyd_safe1	2018	-0.1552	0.0358	7904	-0.2252	-0.0851	-4.3403	**1.44E-05**
(PC1_hyd_safe-1) - PC1_hyd_safe1	2019	-0.1832	0.0358	7886	-0.2535	-0.1130	-5.1119	**3.26E-07**
(PC1_hyd_safe-1) - PC1_hyd_safe1	2020	-0.2710	0.0359	7826	-0.3414	-0.2007	-7.5554	**4.65E-14**
(PC1_hyd_safe-1) - PC1_hyd_safe1	2021	-0.1530	0.0361	7858	-0.2237	-0.0823	-4.2420	**2.24E-05**
(PC2_stom_con-1) - PC2_stom_con1	2016	0.0087	0.0328	10428	-0.0555	0.0730	0.2666	7.90E-01
(PC2_stom_con-1) - PC2_stom_con1	2017	0.0857	0.0324	10381	0.0223	0.1492	2.6497	**8.07E-03**
(PC2_stom_con-1) - PC2_stom_con1	2018	-0.0025	0.0325	10381	-0.0661	0.0612	-0.0763	9.39E-01
(PC2_stom_con-1) - PC2_stom_con1	2019	-0.0469	0.0327	10373	-0.1111	0.0172	-1.4345	1.51E-01
(PC2_stom_con-1) - PC2_stom_con1	2020	-0.0774	0.0333	10368	-0.1428	-0.0121	-2.3238	**2.02E-02**
(PC2_stom_con-1) - PC2_stom_con1	2021	0.0332	0.0338	10390	-0.0331	0.0995	0.9813	3.26E-01

Supplementary Figures

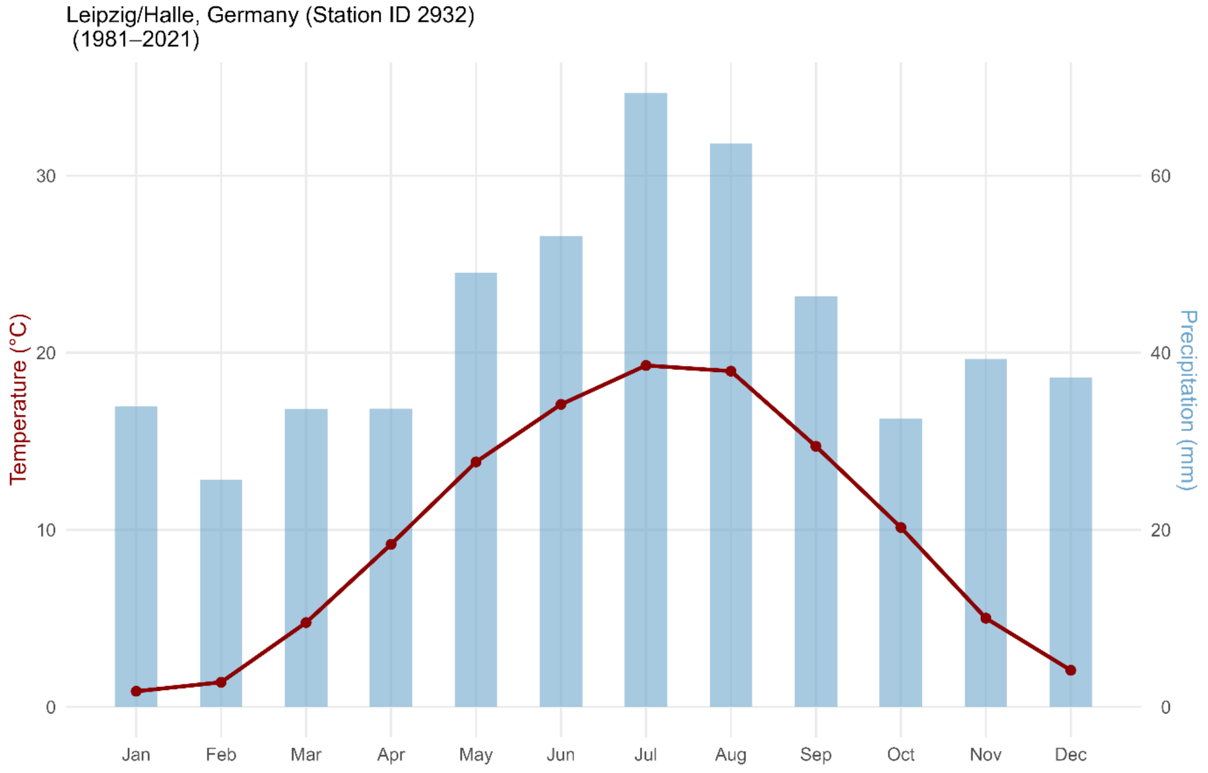

Fig. S 1: ***Climate diagram*** *(Walter–Lieth type) for Halle/Leipzig, Germany (51°26'N, 12°14'E; Station ID 2932) with mean monthly temperature (red line) and precipitation (blue bars) based on 1981–2021 monthly mean values. The diagram follows the classical convention where 1 °C corresponds to 2 mm precipitation. Data source: Deutscher Wetterdienst (DWD).*

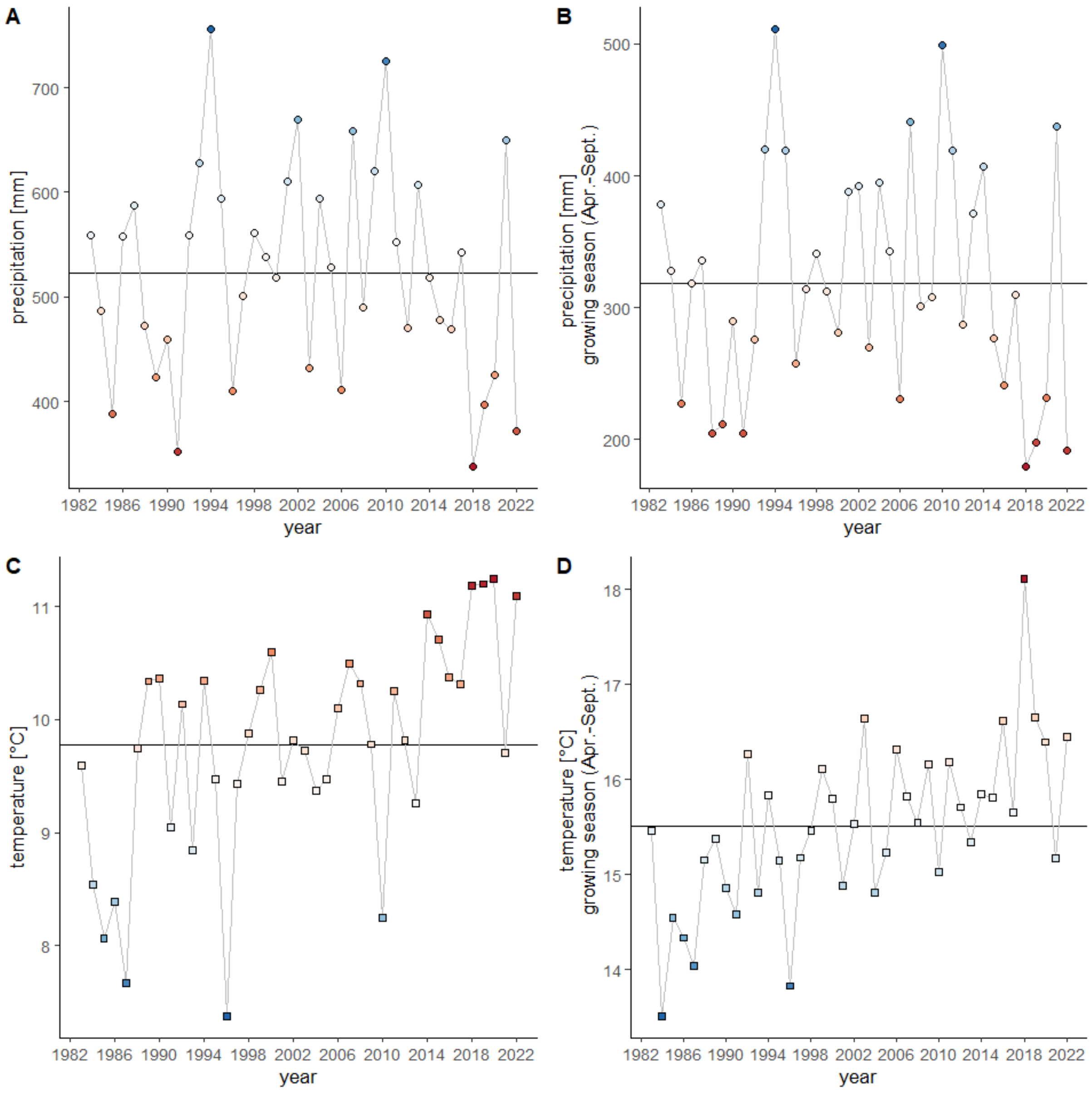

Fig. S 2: ***Precipitation and Temperature.*** *Sum of precipitation and mean temperature in Leipzig/Halle from 1982 to 2022 for the whole year (A, C) and in the growing season (April-September; B, D). The horizontal line indicates the long-term mean over the shown period. (Reprinted from Sachsenmaier et al. (2024))*

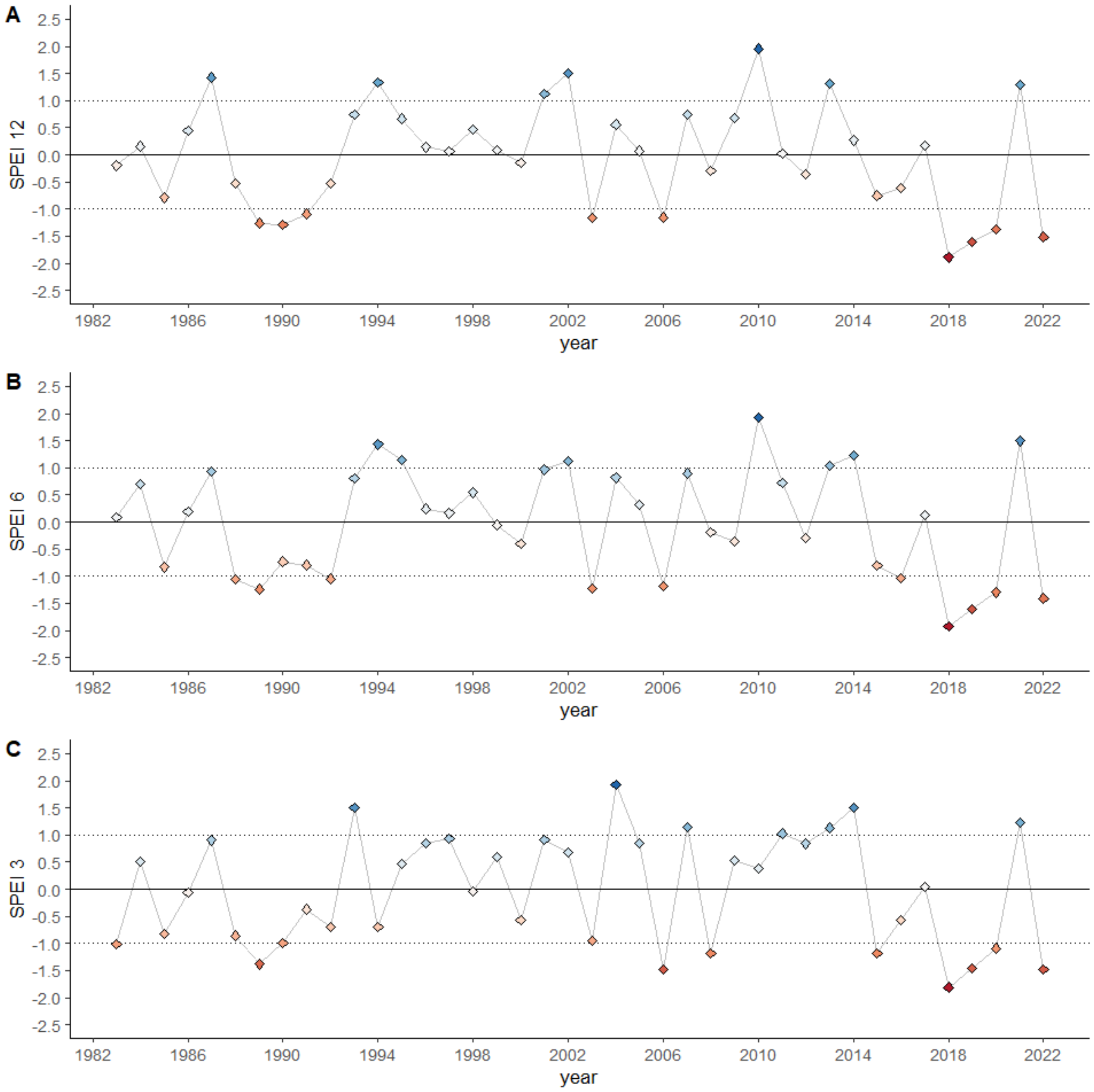

*Fig. S 3: **Standardized Precipitation Evapotranspiration Index** (SPEI) for Leipzig/Halle from 1982 to 2022. Panels show three different time scales of SPEI calculation with (A) January-December (12 months) (B) April-September (6 months; growing season) and (C) May-July (3 months). SPEI values above and below the horizontal dotted lines (>(+1) or <(-1)) are considered as exceptionally wet and dry. The horizontal line at y= 0 represents the long-term mean. (Reprinted from Sachsenmaier et al. (2024))*

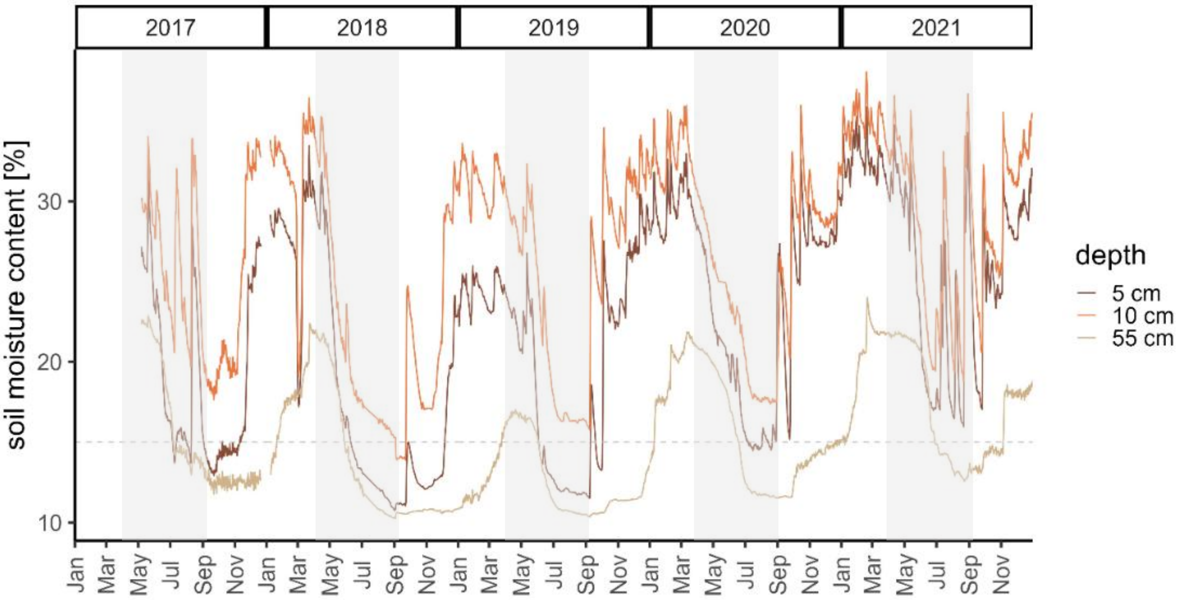

***Fig. S 4: Soil moisture content** [%] of the site since spring 2017. Daily mean values were derived from three different measurement loggers in the MyDiv experiment (at the center of plot 12, plot 57, and plot 77), measuring in 30 min intervals in three different soil depths (5 cm, 10 cm, 55 cm). The dashed horizontal line at 15 % shows the estimated permanent wilting point for the site ((Altermann et al., 2005)). Growing season from April to September is represented by grey background bars.(Reprinted from Sachsenmaier et al. (2024))*

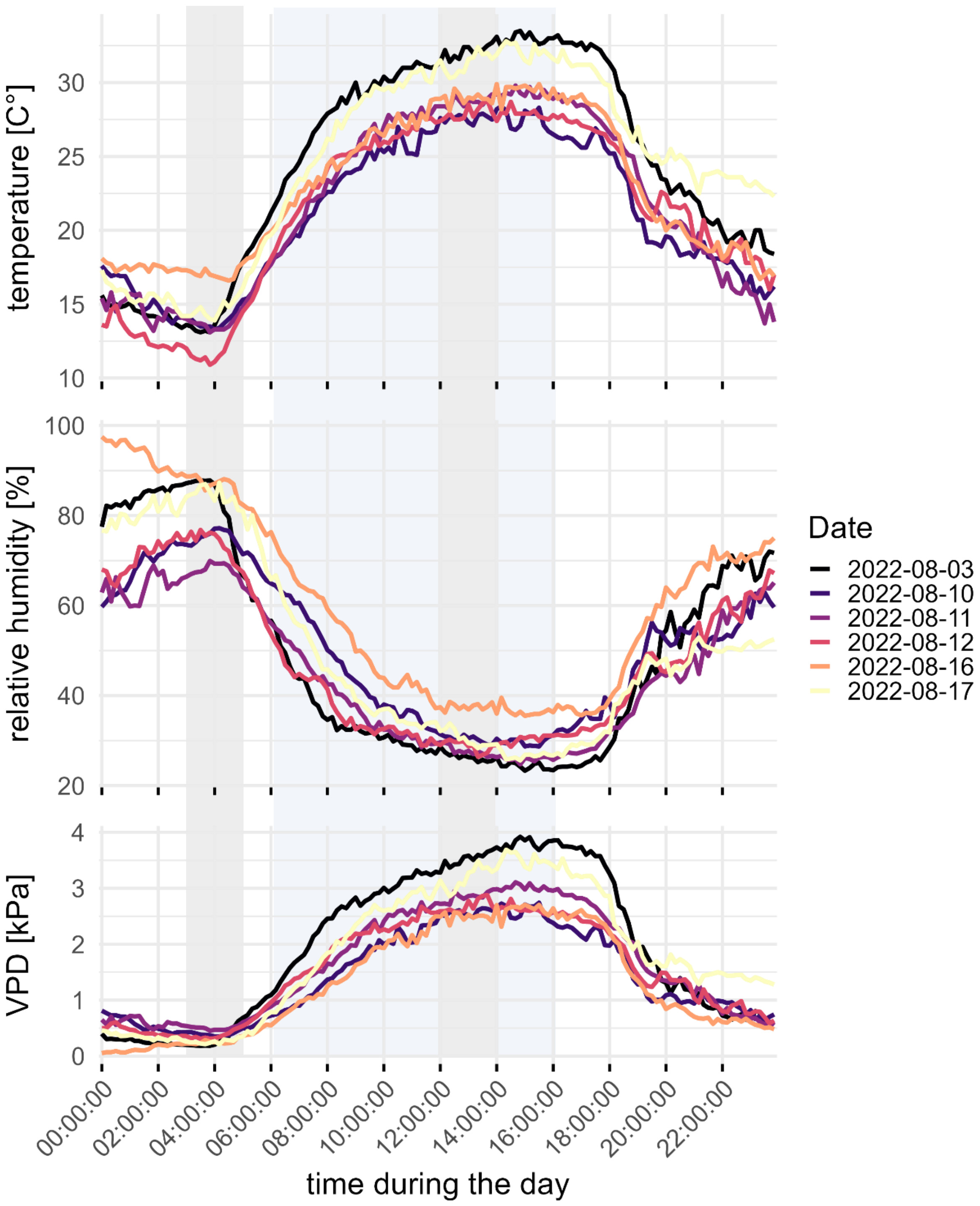

Fig. S 5: Weather conditions during measurement days in summer 2022: *Air temperature in °C, relative humidity in % and vapor pressure deficit (VPD) in kPa over the course of the day. Leaf water potentials were measured at predawn (between 03:00 – 05:00 AM) and midday (between 12:00 PM to 2:00 PM) indicated by grey blocks. Stomatal conductance was measured between 06:00 AM and 4:00 PM (except during 12:00–2:00 PM due to water potential measurements) which are highlighted with a blue layer. Data from the German Weather Station Network (DWD), recorded at the Bad Lauchstädt station (Station ID: 02878) located at 51.3909°N, 11.8786°E.*

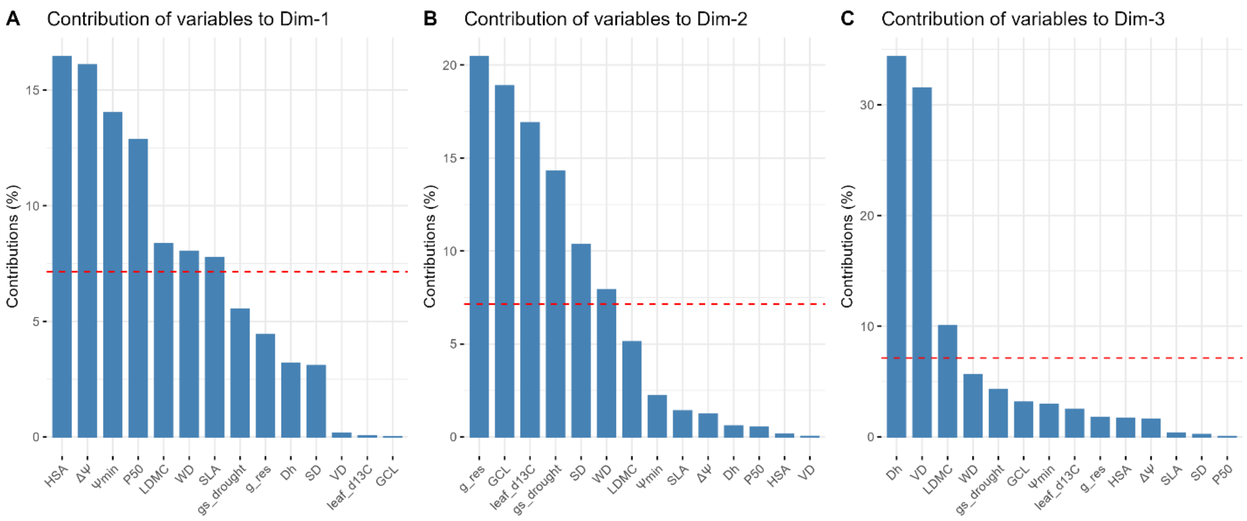

__Fig. S 6: Contributions of variables to the first three principal components (PCs) from the PCA analysis__ (in percentage of the total variance explained). (A) Contributions of variables to PC1, (B) contributions of variables to PC2, and (C) contributions of variables to PC3. The red line indicates the expected average contribution if all variables contributed equally to the respective principal component, i.e. variables exceeding the red line contribute more than the average to the respective PC.

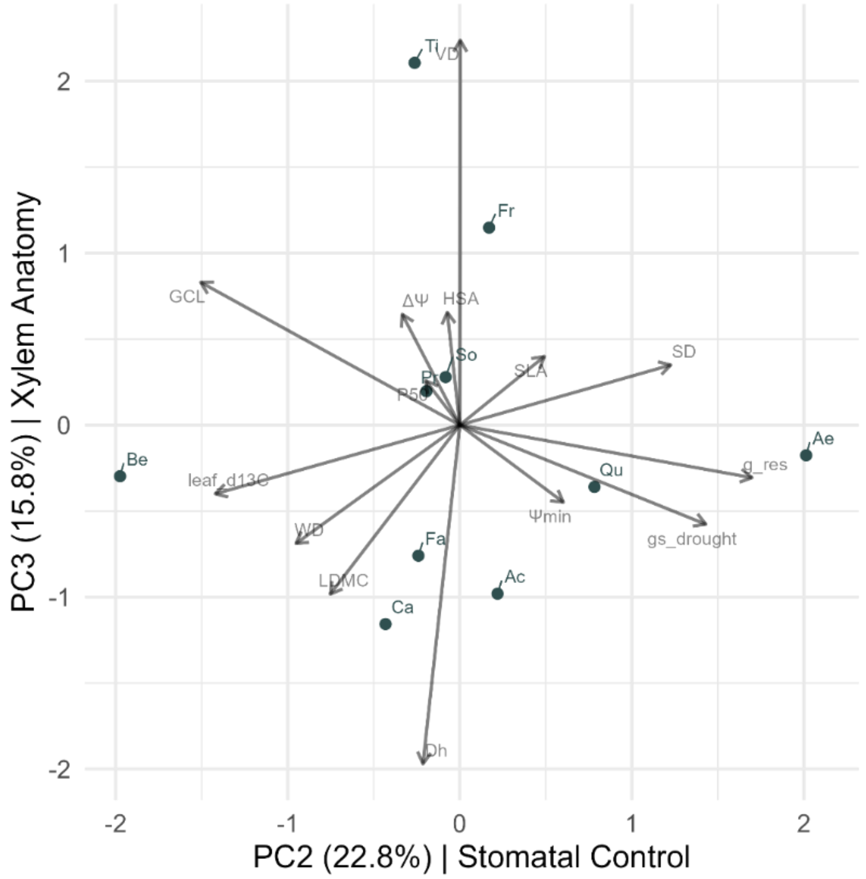

__Fig. S 7: Biplot of PC 2 and PC 3 of the Principal Component Analysis (PCA):__ The third principal component (PC3) represents a gradient between high vessel density (VD) and high hydraulically weighted vessel diameter (D_h), and is orthogonal to PC2 which reflects stomatal control.

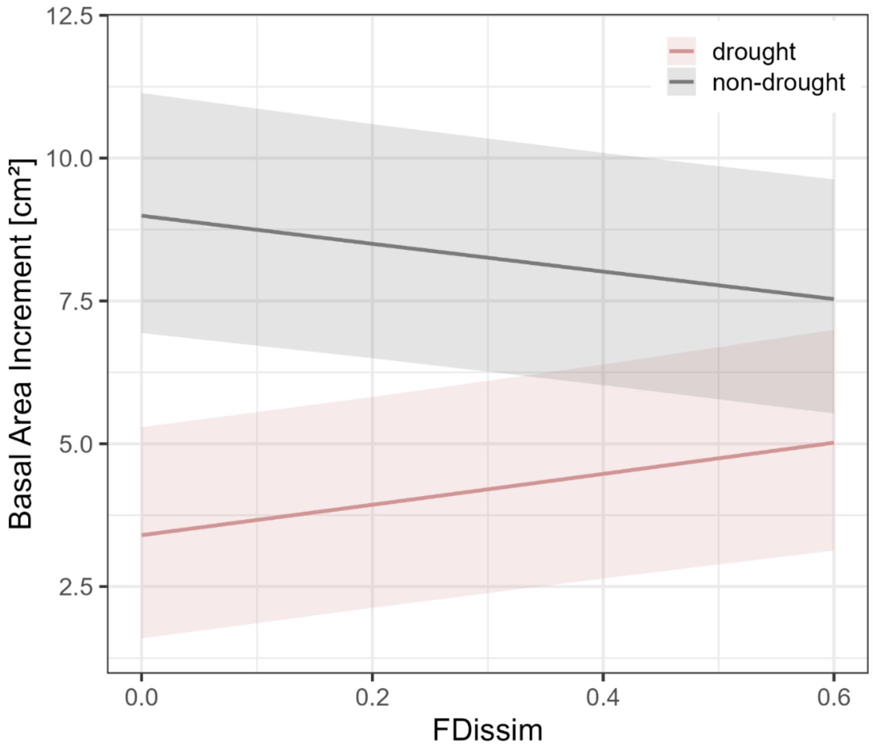

Fig. S 8: The effect of functional dissimilarity to the neighbourhood (FDissim) on tree growth *(as basal area increment),* ***separated by drought and non-drought years****. Lines show model predictions from a linear mixed-effects model including tree size and competition index as covariates, with random effects for year and plot/tree ID. Shaded areas represent 95% confidence intervals around the predictions. Colors indicate drought status: red shades correspond to drought years (2018–2020), gray shades to non-drought years (2016, 2017, 2021). The model explained 56% and 64% of growth variation through its fixed (marginal R2) and fixed and random effects (conditional R2).*

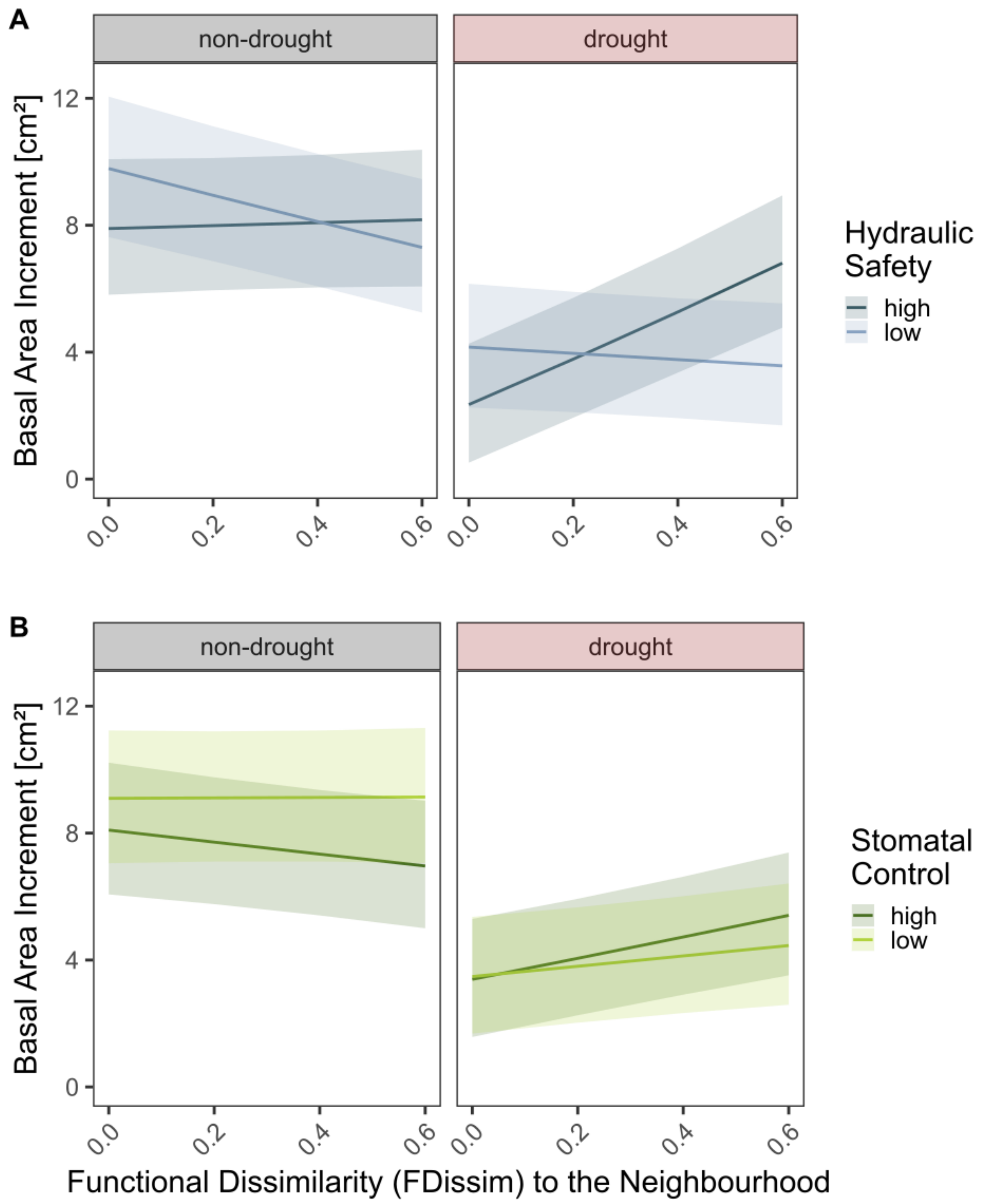

***Fig. S 9: The effect of functional dissimilarity to the neighbourhood (FDissim) in interaction with hydraulic safety (A) or stomatal control (B)** on tree growth (as basal area increment), **separated by drought and non-drought years**. Lines show model predictions from a linear mixed-effects model including tree size and competition index as covariates, with random effects for year and plot/tree ID. Shaded areas represent 95% confidence intervals around the predictions. The panel non-drought corresponds to non-drought years (2016, 2017, 2021) and the panel drought corresponds to drought years (2018–2020). The models explained 56% and 65% (A) and 57 % and 65 % (B) of growth variation through its fixed (marginal R2) and fixed and random effects (conditional R2).*

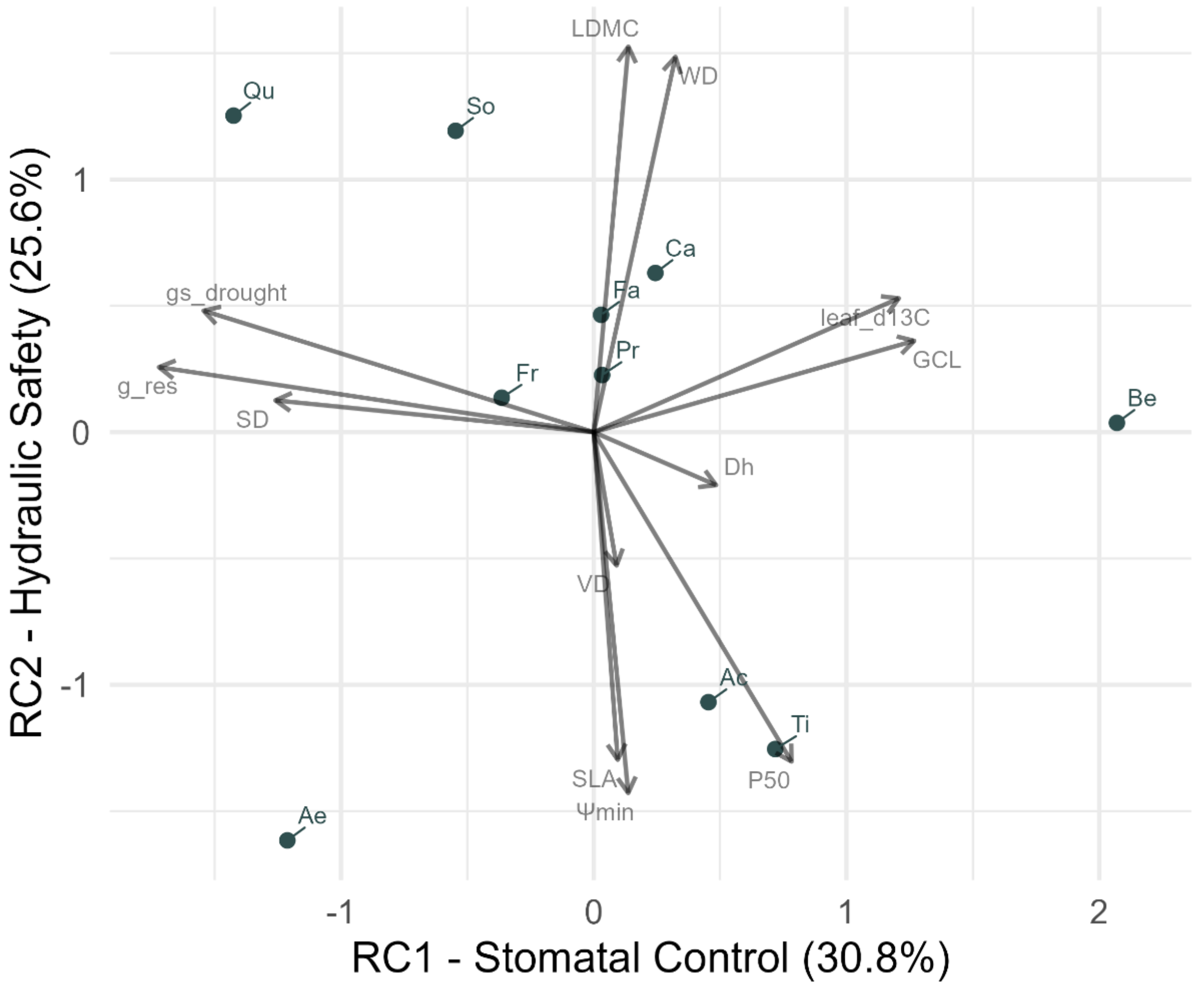

***Fig. S 10: Principal Component Analysis (PCA) after removing two traits related to isohydry**: hydroscape area (HSA) and ΔΨ. The original PC1 now corresponds to the second rotated component (RC2) and PC2 to RC1. Despite the change in axis ordering, the main trait associations and ecological gradients remain consistent, compared to the original PCA in Fig. 2 (see main text, results).*

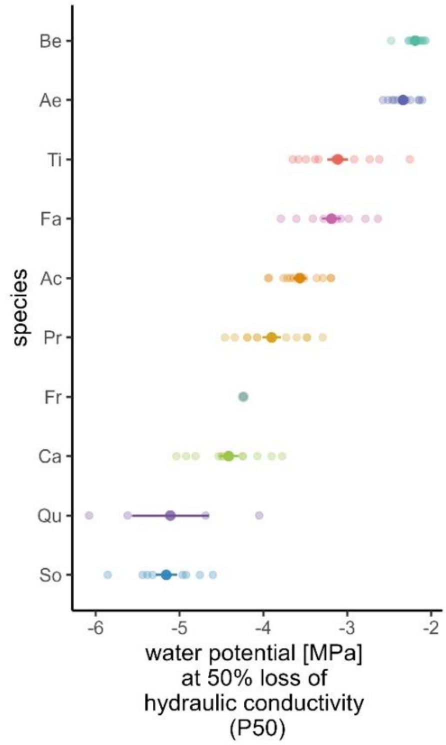

Fig. S 11: Water potential at 50 % loss of hydraulic conductivity (P50) *in MPa. For diffuse-porous species, vulnerability curves were measured at branch level via the Cavitron method, for ring-porous species, they were measured at the leaf level using the Cavicam method (see Methods and Tab. S1 for porosity).*

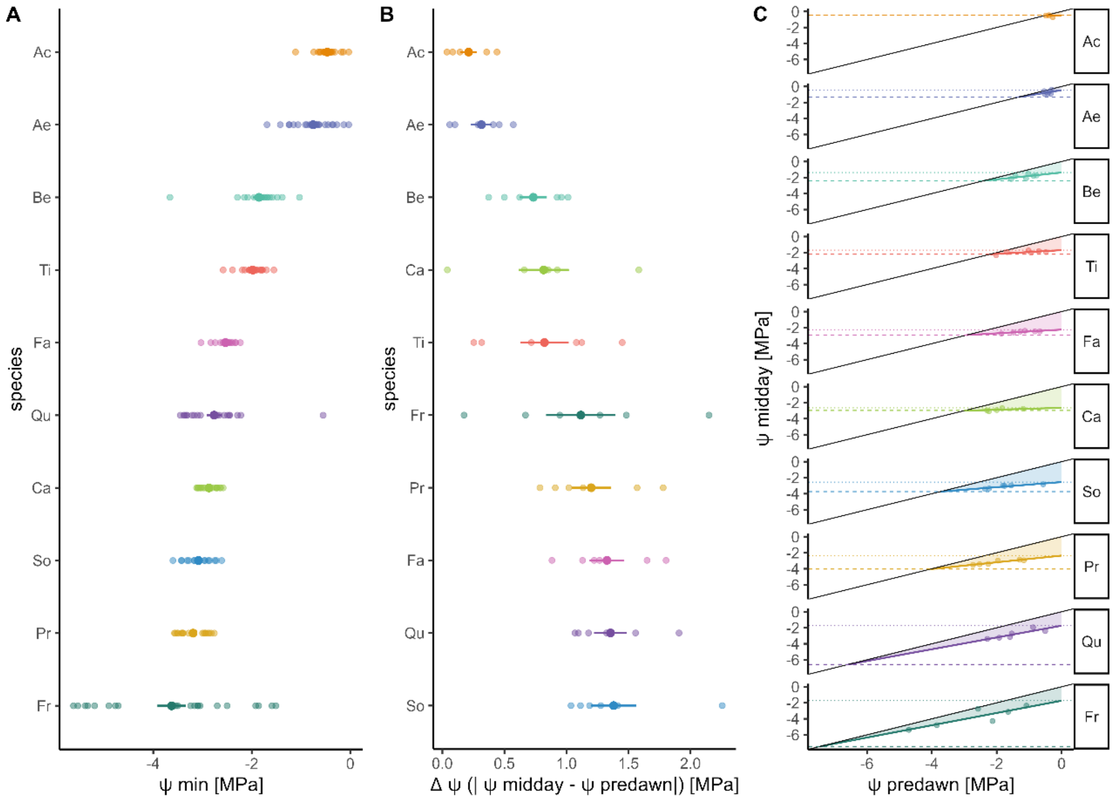

Fig. S 12: Water potential traits*: (A) minimum leaf water potential in MPa, (B) delta between midday and predawn water potential in MPa, (C) Hydroscape area, shown as coloured area between the regression line between midday and predawn water potential and the 1:1 line (Meinzer et al., 2016). Solid circles with error bars represent species means ± standard deviation. For species abbreviations, see Tab. S1.*

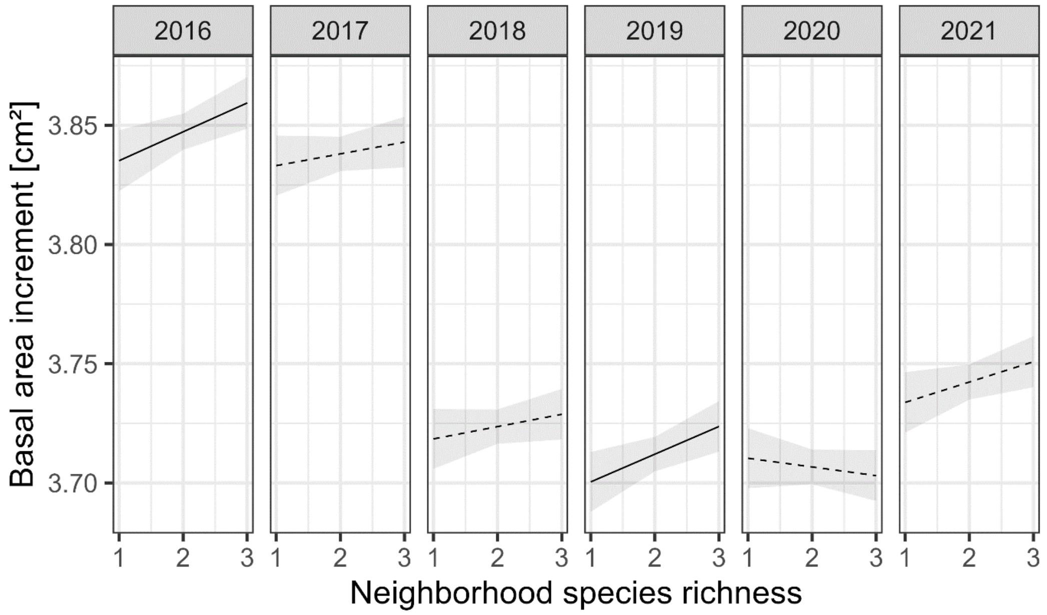

Fig. S 13: Neighbourhood species richness as a predictor of individual tree growth across years. *Regression lines and confidence intervals (95%) show linear mixed-effects model fits that predict growth as basal area increment (log-transformed) based on the interactive effects of neighbourhood species richness, while controlling for tree size and competition as additional fixed effects. The random effects structure includes the tree ID nested within experimental plot ID. Slopes significantly different from zero (as determined by* emtrends*) are represented with solid lines, while non-significant slopes are shown with dashed lines. The model explained 55% and 59% of the variation in growth through its fixed (marginal R2) and fixed and random effects (conditional R2).*

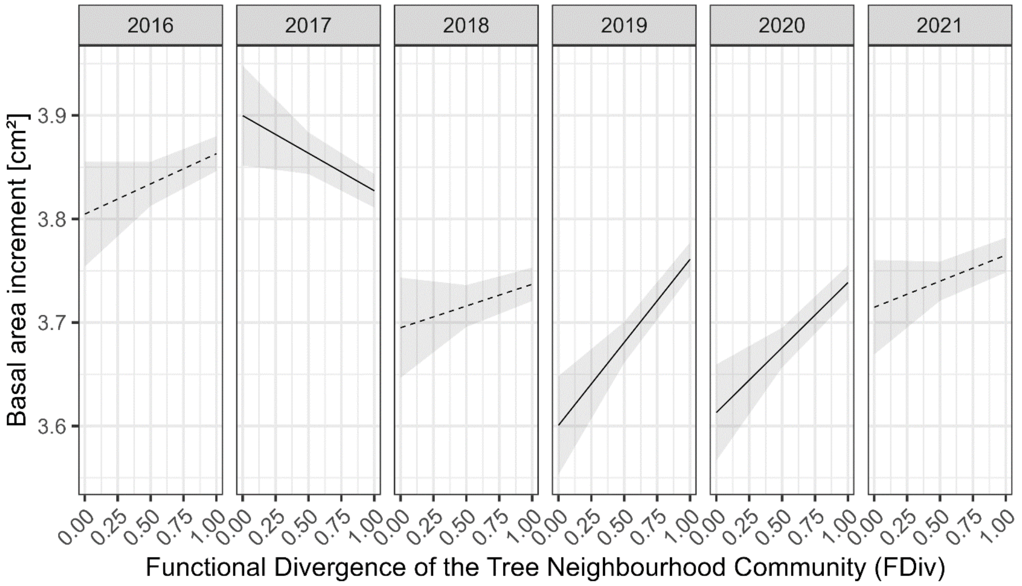

Fig. S 14: The effect of Functional Divergence of the Tree Neighbourhood Community (FDiv) on tree growth in the four-species mixtures of the experiment. *Regression lines and confidence intervals (95% CIs) show linear mixed-effects model fits that predict growth as basal area increment (log-transformed) based on the interactive effects of FDiv and year, while controlling for tree size and competition as additional fixed effects. The random effect structure includes the tree ID nested within experimental plot ID. The model explained 65,5 % and 62,0 % of the variation in growth through its fixed (marginal R2) and fixed and random effects (conditional R2). Slopes significantly different from zero (as determined by the function emtrends() from the emmeans package in R) are represented with solid lines, while non-significant slopes are shown with dashed lines. Note: This analysis was conducted as a comparison with the Functional Dissimilarity (FDissim) model. To maintain a similar model structure, we used the same fixed and random effects. However, because FDiv can only be calculated for a minimum of 3 species, the baseline dataset was smaller and only included the four-species mixtures.*

References

Altermann, M., Rinklebe, J., Merbach, I., Körschens, M., Langer, U., & Hofmann, B. (2005). Chernozem—Soil of the Year 2005. *Journal of Plant Nutrition and Soil Science*, *168*(6), 725–740. https://doi.org/10.1002/jpln.200521814

Avila, R. T., Kane, C. N., Batz, T. A., Trabi, C., Damatta, F. M., Jansen, S., & McAdam, S. A. M. (2023). The relative area of vessels in xylem correlates with stem embolism resistance within and between genera. *Tree Physiology*, *43*(1), 75–87. https://doi.org/10.1093/treephys/tpac110

Baca Cabrera, J. C., Vanderborght, J., Couvreur, V., Behrend, D., Gaiser, T., Nguyen, T. H., & Lobet, G. (2024). Root hydraulic properties: An exploration of their variability across scales. *Plant Direct*, *8*(4), e582. https://doi.org/10.1002/pld3.582

Blackman, C. J., Pfautsch, S., Choat, B., Delzon, S., Gleason, S. M., & Duursma, R. A. (2016). Toward an index of desiccation time to tree mortality under drought. *Plant, Cell & Environment*, *39*(10), 2342–2345. https://doi.org/10.1111/pce.12758

Brodribb, T. J., Holbrook, N. M., Edwards, E. J., & Gutiérrez, M. V. (2003). Relations between stomatal closure, leaf turgor and xylem vulnerability in eight tropical dry forest trees. *Plant, Cell & Environment*, *26*(3), 443–450. https://doi.org/10.1046/j.1365-3040.2003.00975.x

Brodribb, T. J., McAdam, S. A. M., Jordan, G. J., & Martins, S. C. V. (2014). Conifer species adapt to low-rainfall climates by following one of two divergent pathways. *Proceedings of the National Academy of Sciences of the United States of America*, *111*(40), 14489–14493. https://doi.org/10.1073/pnas.1407930111

Brodribb, T. J., Skelton, R. P., McAdam, S. A. M., Bienaimé, D., Lucani, C. J., & Marmottant, P. (2016). Visual quantification of embolism reveals leaf vulnerability to hydraulic failure. *New Phytologist*, *209*(4), 1403–1409. https://doi.org/10.1111/nph.13846

Cernusak, L. A., Ubierna, N., Winter, K., Holtum, J. A. M., Marshall, J. D., & Farquhar, G. D. (2013). Environmental and physiological determinants of carbon isotope discrimination in terrestrial plants. *New Phytologist*, *200*(4), 950–965. https://doi.org/10.1111/nph.12423

Cochard, H. (2002). A technique for measuring xylem hydraulic conductance under high negative pressures. *Plant, Cell and Environment*, *25*(6), 815–819. https://doi.org/10.1046/j.1365-3040.2002.00863.x

Cochard, H., Damour, G., Bodet, C., Tharwat, I., Poirier, M., & Améglio, T. (2005). Evaluation of a new centrifuge technique for rapid generation of xylem vulnerability curves. *Physiologia Plantarum*, *124*(4), 410–418. https://doi.org/10.1111/j.1399-3054.2005.00526.x

Cochard, H., Herbette, S., Barigah, T., Badel, E., Ennajeh, M., & Vilagrosa, A. (2010). Does sample length influence the shape of xylem embolism vulnerability curves? A test with the Cavitron spinning technique. *Plant, Cell and Environment*, *33*(9), 1543–1552. https://doi.org/10.1111/j.1365-3040.2010.02163.x

Comas, L. H., Becker, S. R., Cruz, V. M. V., Byrne, P. F., & Dierig, D. A. (2013). Root traits contributing to plant productivity under drought. *Frontiers in Plant Science*, *4*(NOV), 1–16. https://doi.org/10.3389/fpls.2013.00442

Da Sois, L., Mencuccini, M., Castells, E., Sanchez-Martinez, P., & Martínez-Vilalta, J. (2024). How are physiological responses to drought modulated by water relations and leaf economics' traits in woody plants? *Agricultural Water Management*, *291*, 108613. https://doi.org/10.1016/j.agwat.2023.108613

Duddek, P., Carminati, A., Koebernick, N., Ohmann, L., Lovric, G., Delzon, S., Rodriguez-Dominguez, C. M., King, A., & Ahmed, M. A. (2022). The impact of drought-induced root and root hair shrinkage on root–soil contact. *Plant Physiology*, *189*(3), 1232–1236. https://doi.org/10.1093/plphys/kiac144

Duursma, R. A., Blackman, C. J., Lopéz, R., Martin-StPaul, N. K., Cochard, H., & Medlyn, B. E. (2019). On the minimum leaf conductance: Its role in models of plant water use, and ecological and environmental controls. *New Phytologist*, *221*(2), 693–705. https://doi.org/10.1111/nph.15395

Eller, C. B., de Barros, F. V., Bittencourt, P. R. L., Rowland, L., Mencuccini, M., & Oliveira, R. S. (2018). Xylem hydraulic safety and construction costs determine tropical tree growth. *Plant, Cell & Environment*, *41*(3), 548–562. https://doi.org/10.1111/pce.13106

Fan, D.-Y., Jie, S.-L., Liu, C.-C., Zhang, X.-Y., Xu, X.-W., Zhang, S.-R., & Xie, Z.-Q. (2011). The trade-off between safety and efficiency in hydraulic architecture in 31 woody species in a karst area. *Tree Physiology*, *31*(8), 865–877. https://doi.org/10.1093/treephys/tpr076

Fu, X., & Meinzer, F. C. (2019). Metrics and proxies for stringency of regulation of plant water status (iso/anisohydry): A global data set reveals coordination and trade-offs among water transport traits. *Tree Physiology*, *39*(1), 122–134. https://doi.org/10.1093/treephys/tpy087

Gauthey, A., Peters, J. M. R., Carins-Murphy, M. R., Rodriguez-Dominguez, C. M., Li, X., Delzon, S., King, A., López, R., Medlyn, B. E., Tissue, D. T., Brodribb, T. J., & Choat, B. (2020). Visual and hydraulic techniques produce similar estimates of cavitation resistance in woody species. *New Phytologist*, *228*(3), 884–897. https://doi.org/10.1111/nph.16746

Guillemot, J., Martin-StPaul, N. K., Bulascoschi, L., Poorter, L., Morin, X., Pinho, B. X., le Maire, G., R. L. Bittencourt, P., Oliveira, R. S., Bongers, F., Brouwer, R., Pereira, L., Gonzalez Melo, G. A., Boonman, C. C. F., Brown, K. A., Cerabolini, B. E. L., Niinemets, Ü., Onoda, Y., Schneider, J. V., … Brancalion, P. H. S. (2022). Small and slow is safe: On the drought tolerance of tropical tree species. *Global Change Biology*, *28*(8), 2622–2638. https://doi.org/10.1111/gcb.16082

Isasa, E., Link, R. M., Jansen, S., Tezeh, F. R., Kaack, L., Sarmento Cabral, J., & Schuldt, B. (2023). Addressing controversies in the xylem embolism resistance–vessel diameter relationship. *New Phytologist*, *238*(1), 283–296. https://doi.org/10.1111/nph.18731

Jaeger, F. C., Handa, I. T., Paquette, A., Parker, W. C., & Messier, C. (2024). Young temperate tree species show different fine root acclimation capacity to growing season water availability. *Plant and Soil*, *496*(1), 485–504. https://doi.org/10.1007/s11104-023-06377-w

Kahmen, A., Basler, D., Hoch, G., Link, R. M., Schuldt, B., Zahnd, C., & Arend, M. (2022). Root water uptake depth determines the hydraulic vulnerability of temperate European tree species

during the extreme 2018 drought. *Plant Biology*, *24*(7), 1224–1239. https://doi.org/10.1111/plb.13476

Kattge, J., Bönisch, G., Díaz, S., Lavorel, S., Prentice, I. C., Leadley, P., Tautenhahn, S., Werner, G. D. A., Aakala, T., Abedi, M., Acosta, A. T. R., Adamidis, G. C., Adamson, K., Aiba, M., Albert, C. H., Alcántara, J. M., Alcázar C, C., Aleixo, I., Ali, H., … Wirth, C. (2020). TRY plant trait database – enhanced coverage and open access. *Global Change Biology*, *26*(1), 119–188. https://doi.org/10.1111/gcb.14904

Klein, T. (2014). The variability of stomatal sensitivity to leaf water potential across tree species indicates a continuum between isohydric and anisohydric behaviours. *Functional Ecology*, *28*(6), 1313–1320. https://doi.org/10.1111/1365-2435.12289

Kröber, W., Zhang, S., Ehmig, M., & Bruelheide, H. (2014). Linking Xylem Hydraulic Conductivity and Vulnerability to the Leaf Economics Spectrum—A Cross-Species Study of 39 Evergreen and Deciduous Broadleaved Subtropical Tree Species. *PLoS ONE*, *9*(11), e109211–e109211. https://doi.org/10.1371/journal.pone.0109211

Lehto, T., & Zwiazek, J. J. (2011). Ectomycorrhizas and water relations of trees: A review. *Mycorrhiza*, *21*(2), 71–90. https://doi.org/10.1007/s00572-010-0348-9

Leuschner, C., Wedde, P., & Lübbe, T. (2019). The relation between pressure–volume curve traits and stomatal regulation of water potential in five temperate broadleaf tree species. *Annals of Forest Science*, *76*(2), 60–60. https://doi.org/10.1007/s13595-019-0838-7

Lv, C., Wang, C., Li, Y., & Zhou, Z. (2023). Coordination among root exudation C, mycorrhizal colonization, and functional traits and their responses to drought in five temperate tree species. *Forest Ecology and Management*, *546*, 121316. https://doi.org/10.1016/j.foreco.2023.121316

Machado, R., Loram-Lourenço, L., Farnese, F. S., Alves, R. D. F. B., de Sousa, L. F., Silva, F. G., Filho, S. C. V., Torres-Ruiz, J. M., Cochard, H., & Menezes-Silva, P. E. (2021). Where do leaf water leaks come from? Trade-offs underlying the variability in minimum conductance across tropical

savanna species with contrasting growth strategies. *New Phytologist*, *229*(3), 1415–1430. https://doi.org/10.1111/nph.16941

Maherali, H., Moura, C. F., Caldeira, M. C., Willson, C. J., & Jackson, R. B. (2006). Functional coordination between leaf gas exchange and vulnerability to xylem cavitation in temperate forest trees. *Plant, Cell & Environment*, *29*(4), 571–583. https://doi.org/10.1111/j.1365-3040.2005.01433.x

Martínez-Vilalta, J., & Garcia-Forner, N. (2017). Water potential regulation, stomatal behaviour and hydraulic transport under drought: Deconstructing the iso/anisohydric concept. *Plant, Cell & Environment*, *40*(6), 962–976. https://doi.org/10.1111/pce.12846

Martin-StPaul, N. K., Delzon, S., & Cochard, H. (2017). Plant resistance to drought depends on timely stomatal closure. *Ecology Letters*, *20*(11), 1437–1447. https://doi.org/10.1111/ele.12851

McDowell, N., Pockman, W. T., Allen, C. D., Breshears, D. D., Cobb, N., Kolb, T., Plaut, J., Sperry, J., West, A., Williams, D. G., & Yepez, E. A. (2008). Mechanisms of plant survival and mortality during drought: Why do some plants survive while others succumb to drought? *New Phytologist*, *178*(4), 719–739. https://doi.org/10.1111/j.1469-8137.2008.02436.x

Meinzer, F. C., Woodruff, D. R., Marias, D. E., Smith, D. D., McCulloh, K. A., Howard, A. R., & Magedman, A. L. (2016). Mapping 'hydroscapes' along the iso- to anisohydric continuum of stomatal regulation of plant water status. *Ecology Letters*, *19*(11), 1343–1352. https://doi.org/10.1111/ele.12670

Münchinger, I. K., Hajek, P., Akdogan, B., Caicoya, A. T., & Kunert, N. (2023). Leaf thermal tolerance and sensitivity of temperate tree species are correlated with leaf physiological and functional drought resistance traits. *Journal of Forestry Research*, *34*(1), 63–76. https://doi.org/10.1007/s11676-022-01594-y

Nye, P. H. (1994). The effect of root shrinkage on soil water inflow. *Philosophical Transactions of the Royal Society of London. Series B: Biological Sciences*, *345*(1314), 395–402. https://doi.org/10.1098/rstb.1994.0117

Ogle, K., Barber, J. J., Willson, C., & Thompson, B. (2009). Hierarchical statistical modeling of xylem vulnerability to cavitation. *New Phytologist*, *182*(2), 541–554. https://doi.org/10.1111/j.1469-8137.2008.02760.x

Oliveira, R. S., Eller, C. B., Barros, F. de V., Hirota, M., Brum, M., & Bittencourt, P. (2021). Linking plant hydraulics and the fast–slow continuum to understand resilience to drought in tropical ecosystems. *New Phytologist*, *230*(3), 904–923. https://doi.org/10.1111/nph.17266

Pammenter, N. W., & van der Willigen, C. (1998). Mathematical and statistical analysis of the curves illustrating vulnerability of xylem to cavitation. *Tree Physiology*, *18*(8–9). https://academic.oup.com/treephys/article-abstract/18/8-9/589/1632591?redirectedFrom=fulltext

Petrík, P., Petek-Petrík, A., Lamarque, L. J., Link, R. M., Waite, P.-A., Ruehr, N. K., Schuldt, B., & Maire, V. (2024). Linking stomatal size and density to water use efficiency and leaf carbon isotope ratio in juvenile and mature trees. *Physiologia Plantarum*, *176*(6), e14619. https://doi.org/10.1111/ppl.14619

Pya, N. (2025). *scam: Shape Constrained Additive Models* (Version 1.2-18) [Computer software]. https://cran.r-project.org/web/packages/scam/index.html

Reich, P. B. (2014). The world-wide ‘fast-slow’ plant economics spectrum: A traits manifesto. *Journal of Ecology*, *102*(2), 275–301. https://doi.org/10.1111/1365-2745.12211

Sachsenmaier, L., Schnabel, F., Dietrich, P., Eisenhauer, N., Ferlian, O., Quosh, J., Richter, R., & Wirth, C. (2024). Forest growth resistance and resilience to the 2018–2020 drought depend on tree diversity and mycorrhizal type. *Journal of Ecology*, *112*(8), 1787–1803. https://doi.org/10.1111/1365-2745.14360

Schnabel, F., Barry, K. E., Eckhardt, S., Guillemot, J., Geilmann, H., Kahl, A., Moossen, H., Bauhus, J., & Wirth, C. (2024). Neighbourhood species richness and drought-tolerance traits modulate tree growth and δ13C responses to drought. *Plant Biology*, 1–16. https://doi.org/10.1111/plb.13611

Schuldt, B., Ahmed, M., Choat, B., Delzon, S., Jansen, S., Kotowska, M. M., Leuschner, C., Lamarque, L. J., Lübbe, T., Petrik, P., Torres-Ruiz, J. M., & McAdam, S. (2025). *Revisiting Paradigms Related to Root Hydraulic Limitation Under Drought* (pp. 1–30). Springer. https://doi.org/10.1007/124_2025_93

Schumann, K., Leuschner, C., & Schuldt, B. (2019). Xylem hydraulic safety and efficiency in relation to leaf and wood traits in three temperate Acer species differing in habitat preferences. *Trees*, *33*(5), 1475–1490. https://doi.org/10.1007/s00468-019-01874-x

Sperry, J. S., Hacke, U. G., Oren, R., & Comstock, J. P. (2002). Water deficits and hydraulic limits to leaf water supply. *Plant, Cell & Environment*, *25*(2), 251–263. https://doi.org/10.1046/j.0016-8025.2001.00799.x

Sperry, J. S., Nichols, K. L., Sullivan, J. E. M., & Eastlack, S. E. (1994). Xylem Embolism in Ring-Porous, Diffuse-Porous, and Coniferous Trees of Northern Utah and Interior Alaska. *Ecology*, *75*(6), 1736–1752. https://doi.org/10.2307/1939633

Vasseur, F., Cornet, D., Beurier, G., Messier, J., Rouan, L., Bresson, J., Ecarnot, M., Stahl, M., Heumos, S., Gérard, M., Reijnen, H., Tillard, P., Lacombe, B., Emanuel, A., Floret, J., Estarague, A., Przybylska, S., Sartori, K., Gillespie, L. M., … Violle, C. (2022). A Perspective on Plant Phenomics: Coupling Deep Learning and Near-Infrared Spectroscopy. *Frontiers in Plant Science*, *13*. https://doi.org/10.3389/fpls.2022.836488

Waite, P.-A., Kumar, M., Link, R. M., & Schuldt, B. (2024). Coordinated hydraulic traits influence the two phases of time to hydraulic failure in five temperate tree species differing in stomatal stringency. *Tree Physiology*, *44*(5), tpae038. https://doi.org/10.1093/treephys/tpae038

White, F. M. (1991). *Viscous fluid flow* (2nd ed). McGraw-Hill.

Wood, S. (2025). *mgcv: Mixed GAM Computation Vehicle with Automatic Smoothness Estimation* (Version 1.9-3) [Computer software]. https://cran.r-project.org/web/packages/mgcv/index.html

Zanne, A. E., Westoby, M., Falster, D. S., Ackerly, D. D., Loarie, S. R., Arnold, S. E. J., & Coomes, D. A. (2010). Angiosperm wood structure: Global patterns in vessel anatomy and their relation to wood density and potential conductivity. *American Journal of Botany*, *97*(2), 207–215. https://doi.org/10.3732/ajb.0900178

Ziemińska, K., Rosa, E., Gleason, S. M., & Holbrook, N. M. (2020). Wood day capacitance is related to water content, wood density, and anatomy across 30 temperate tree species. *Plant, Cell & Environment*, *43*(12), 3048–3067. https://doi.org/10.1111/pce.13891
